# A novel nematode effector suppresses plant immunity by activating host reactive oxygen species‐scavenging system

**DOI:** 10.1111/nph.13701

**Published:** 2015-10-20

**Authors:** Borong Lin, Kan Zhuo, Shiyan Chen, Lili Hu, Longhua Sun, Xiaohong Wang, Lian‐Hui Zhang, Jinling Liao

**Affiliations:** ^1^ Laboratory of Plant NematologySouth China Agricultural UniversityGuangzhou510642China; ^2^ Guangdong Province Key Laboratory of Microbial Signals and Disease ControlSouth China Agricultural UniversityGuangzhou510642China; ^3^ School of Integrative Plant ScienceCornell UniversityIthacaNY14853USA; ^4^ Robert W. Holley Center for Agriculture and HealthUS Department of AgricultureAgricultural Research ServiceIthacaNY14853USA; ^5^ Institute of Molecular and Cell Biology61 Biopolis DriveSingapore138673Singapore; ^6^ Guangdong Vocational College of Ecological EngineeringGuangzhou510520China

**Keywords:** *Arabidopsis thaliana*, ferredoxin : thioredoxin reductase catalytic subunit, *Meloidogyne javanica*, pathogen‐associated molecular pattern (PAMP)‐triggered immunity (PTI), reactive oxygen species (ROS), transthyretin‐like protein (TTL)

## Abstract

- Evidence is emerging that plant‐parasitic nematodes can secrete effectors to interfere with the host immune response, but it remains unknown how these effectors can conquer host immune responses. Here, we depict a novel effector, MjTTL5, that could suppress plant immune response.
- Immunolocalization and transcriptional analyses showed that MjTTL5 is expressed specifically within the subventral gland of *Meloidogyne javanica* and up‐regulated in the early parasitic stage of the nematode. Transgenic Arabidopsis lines expressing *MjTTL5* were significantly more susceptible to *M. javanica* infection than wild‐type plants, and vice versa, *in planta* silencing of MjTTL5 substantially increased plant resistance to *M. javanica*.
- Yeast two‐hybrid, coimmunoprecipitation and bimolecular fluorescent complementation assays showed that MjTTL5 interacts specifically with Arabidopsis ferredoxin : thioredoxin reductase catalytic subunit (AtFTRc), a key component of host antioxidant system. The expression of AtFTRc is induced by the infection of *M. javanica*. Interaction between AtFTRc and MjTTL could drastically increase host reactive oxygen species‐scavenging activity, and result in suppression of plant basal defenses and attenuation of host resistance to the nematode infection.
- Our results demonstrate that the host ferredoxin : thioredoxin system can be exploited cunningly by *M. javanica*, revealing a novel mechanism utilized by plant–parasitic nematodes to subjugate plant innate immunity and thereby promoting parasitism.

## Introduction

Root‐knot nematodes (RKNs), that is *Meloidogyne* spp., are one family of the most devastating plant‐parasitic nematodes (PPNs), infecting > 3000 plant species from diverse plant families worldwide, which results in *c*. $70 billion worth of economic loss every year (Caboni *et al*., 2012). RKNs are obligate biotrophic nematodes that infect plant roots and get essential nutrients from specialized multinucleate feeding cells known as giant cells. Similar to other soilborne pathogens, RKNs are very hard to control. To develop new and environmentally safe disease control strategies, it is essential to understand their parasitic mechanisms and how they interact with host plants.

Previous studies on bacterial and fungal pathogens have contributed significantly to the understanding of plant defense responses. Plants commonly possess two major types of resistance mechanisms against infection by pathogens and parasites (Jones & Dangl, 2006). The first layer is the general pathogen‐associated molecular pattern (PAMP)‐triggered immunity (PTI) and the second is the more specific effector‐triggered immunity (ETI). The latter is stimulated by plant surveillance proteins (R proteins) upon recognizing specific effector proteins from pathogens (AVR proteins). PTI is a multistep response, which is triggered upon plant pattern recognition receptors (PPRs) recognizing the conserved pathogen molecules. Subsequently, many events are induced, including activation of the mitogen‐activated protein kinase (MAPK) signaling cascade, calcium‐dependent protein kinase (CDPK) and the reactive oxygen species (ROS)‐generating system (Asai *et al*., 2002; Zipfel *et al*., 2004). Given the signaling roles of ROS in triggering various plant defense responses, including activation of defense genes, synthesis of antimicrobial secondary metabolites and strengthening of plant cell walls, and potent toxicity against pathogens, ROS burst has been known as a hallmark of plant innate immunity (Torres, 2010).

The roles of ROS in plant resistance against PPN infection have also been documented. ROS were accumulated at the very early stage of host invasion by *Meloidogyne incognita* and resistant tomato cultivar produced a substantially higher amount of ROS than the susceptible cultivar during *M. incognita* invasion (Melillo *et al*., 2006). Given the important roles of ROS in defense against nematode infection, it is intriguing how PPNs cope with this plant defense response during infection. PPNs are known to secrete a range of effector proteins, produced in the esophageal gland cells, to interfere with various plant processes to facilitate infection and parasitism (Davis *et al*., 2008). These effectors act by degradation and modification of the plant cell wall, regulating plant ubiquitination pathways and interfering with plant signaling pathways (Haegeman *et al*., 2012; Hewezi & Baum, 2013; Mitchum *et al*., 2013). Recently, evidence emerged that PPNs can interfere with host PTI response through secreting effector proteins. The effector Mi‐CRT from the RKN *M. incognita* was shown to suppress the induction of defense marker genes and callose deposition triggered by the PAMP elf18 (Jaouannet *et al*., 2013). Similarly, the GrCEP12 derived from *Globodera rostochiensis* suppresses host PTI responses, including ROS production and the expression of two PTI marker genes triggered by the PAMP flg22 (Chen *et al*., 2013). However, host targets of these two effectors have yet to be identified and the mechanisms with which these effectors suppress PTI responses, including ROS production, remain unclear.

In this study, we report the cloning and characterization of the gene encoding a transthyretin‐like protein designated as MjTTL5 from *M. javanica*. We present several lines of evidence to show that MjTTL5 is an important effector for nematode parasitism. In addition, we show that MjTTL5 plays a role in suppressing host PTI responses. Moreover, we demonstrate that MjTTL5 directly interacts with the Arabidopsis ferredoxin : thioredoxin reductase catalytic subunit (AtFTRc), a key element of the plant antioxidant network (Dos Santos & Rey, 2006), resulting in substantially enhanced plant ROS‐scavenging activity. Together, our data suggest a novel mechanism by which *M. javanica* utilizes its secreted MjTTL5 effector to suppress the oxidative response through the cunning exploitation of the host ROS‐scavenging system.

## Materials and Methods

### Nematode and plant materials

*Meloidogyne javanica* (Treub) Chitwood and *M. incognita* (Kofoid & White) Chitwood were propagated on glasshouse‐grown tomato (*Solanum lycopersicum* Mill, cv Xiahong No. 1). Preparation and hatching of eggs were performed as described previously (Huang *et al*., 2005). *Radopholus similis* Thorne was cultured on excised carrot (*Daucus carota* L.) discs at 25°C (Fallas & Sarah, 1994). Transgenic Arabidopsis (*Arabidopsis thaliana* (L.) Heynh) lines expressing *MjTTL5*, or the AtFTRcpro::GUS lines were generated as described previously (Zhang *et al*., 2006), the AtFTRc T‐DNA mutant line (GK‐686B09) was obtained from the Arabidopsis Information Resource (TAIR) and the homozygous mutant lines were identified by genomic PCR using primers mFTRF/mFTRR/TDNA. The results showed that the two tubes of seed stocks (CS720422 and CS720426) of GK‐686B09 were homozygous (Supporting Information Fig. S1), and these were used for subsequent experiments. Quantitative reverse transcription PCR (RT‐qPCR) was performed to analyze the mRNA abundance of *AtFTRc*. The Arabidopsis ecotype Columbia was used for wild‐type control. The Arabidopsis, tobacco (*Nicotiana benthamiana* L.) and tomato were grown in a glasshouse at 25°C under 16 : 8 h, light : dark conditions.

### Gene amplification and characterization

*Meloidogyne javanica* genomic DNA and total RNA were isolated from fresh hatching preparasitic second‐stage juveniles (pre‐J2s) using the Genomic DNA purification kit (Shenergy Biocolor, Shanghai, China) and TRIzol reagent (Invitrogen), respectively. The *MjTTL5* sequence was obtained using rapid amplification of cDNA ends with the BD SMART cDNA amplification kit (Clontech, Beijing, China) and hiTAIL‐PCR (Liu & Chen, 2007). All primers used in this study were synthesized by Invitrogen Biotechnology Co. Ltd and are listed in Table S1.

The sequence homology of the predicted protein was analyzed using a BLASTx, BLASTn or tBLASTn search of the nonredundant and Expressed Sequence Tags database of the National Center for Biotechnology Information. Sequences were aligned with ClustalW and the signal peptide was predicted using SignalP (Bendtsen *et al*., 2004).

### Southern blot

Ten micrograms of *M. javanica* total genomic DNA were separately digested with *Dpn*I (one cleavage site located in 633–636 bp) and *Sal*I (no cleavage site) before separation by electrophoresis, and transferred to Hybond N^+^ membranes (Amersham‐Biosciences). Probe hybridization and signal detection were performed as described by Lin *et al*. (2013).

### Phylogenetic tree analyses

The deduced amino acid sequences of transthyretin‐like (TTL) homologs were used for Bayesian inference (BI) tree analyses. All proteins used in the BI tree are listed in Table S2. The TRP protein from *Danio rerio* was used as the root of the BI tree. The BI tree was generated according to the method described previously (Kyndt *et al*., 2008).

### Anti‐MjTTL5 polyclonal serum production and analysis

The MjTTL5 protein was purified by affinity chromatography using Ni^2+^ NTA agarose (Qiagen) according to the user manual. The amount and purity of the purified protein were determined by the Bradford method and sodium dodecyl sulfate–polyacrylamide gel electrophoresis (SDS‐PAGE)  (Fig. S2). The protein was used to immunize rabbits intradermally for antiserum production as described previously (Luciano *et al*., 2004).

### Expression analysis

RNA samples were prepared from 100 *M. javanica* nematodes at different life stages as indicated, using the RNA prepmicro kit (Tiangen Biotech, Beijing, China). The cDNA was synthesized using ReverTra Ace qPCR RT Master Mix with gDNA Remover kit (Toyobo, Osaka, Japan). RT‐qPCR was performed using the primer pairs qttlF/qttlR and qactinF/qactinR for amplifying the gene *MjTTL5* and the internal control gene *Mj‐β‐actin* (accession no. AF532605), respectively. RT‐qPCR was performed using the THUNDERBIRD SYBR^®^ qPCR Mix (Toyobo). The relative changes in gene expression were determined using the $2^{-\Delta \Delta C_{T}}$ method (Livak & Schmittgen, 2001).

For immunolocalization analysis of the MjTTL5 on *M. javanica*,* c*. 10 000 freshly hatched J2 were used. Immunolocalization was carried out as described previously (Jaubert *et al*., 2002).

### Interaction analysis

For the yeast two‐hybrid (Y2H) assay, the *MjTTL5* was cloned into pGBKT7 to generate pGBKT7:ttl5 and then transformed into *Saccharomyces cerevisiae* AH109 to generate the bait strain. The Arabidopsis ecotype Columbia cDNA library from roots at 15 d postinfection (dpi) by *M. javanica* was generated in the *S. cerevisiae* strain Y187. Screening for interacting protein and α‐galactosidase (α‐Gal) quantitative assay were performed following the user manual. Other TTL homologs were cloned into the pGBK vector through pEASY‐Uni Seamless Cloning and Assembly Kit (Transgen Biotech, Beijing, China) and cotransformed with the AtFTRc‐pGAD into the AH109.

For coimmunoprecipitation (CoIP) assay, the MjTTL5 and AtFTRc were cloned into the pSPYCE and pSPYNE, respectively. All constructs were confirmed by sequencing and introduced into *Agrobacterium tumefaciens* GV3101. As a control, the pSPYCE‐ttl5 was replaced by pMD1‐green fluorescent protein‐hemagglutinin, and the mixture was also infiltrated in tobacco. At 48 h after infiltration, the proteins were extracted and the CoIP assays were carried out as described previously (Moffett *et al*., 2002).

For the bimolecular fluorescent complementation (BiFC) assay, the transgenic tomato roots expressing AtNTRc‐mCherry were generated as described previously (Ron *et al*., 2014). All constructs (Fig. S3) were purified using the HighPure Maxi Plasmid Kit (Tiangen) and transformed into tomato protoplast through polyethylene glycol as described by Lee *et al*. (2013). Experimental details are given in Methods S1–S3.

### Analysis of AtFTRc expression in different tissues and galls

RNA samples were purified from the leaves, flowers and roots of 30‐d‐old Arabidopsis. The Arabidopsis *AtActin* gene (AT1G49240) was used as an endogenous reference. The relative changes in gene expression between different tissues were determined using the $2^{-\Delta \Delta C_{T}}$ method and relative to expression in the root. To determine *AtFTRc* expression pattern after nematode infection, 100 sterilized *M. javanica* were inoculated into 14‐d‐old Arabidopsis roots. At 2 and 5 dpi, galls, noninfected root tissues and the equivalent part of uninfected control roots were harvested and total RNA or total protein was prepared. For western blot, 5 μg of total proteins from each sample was used. AtFTRc was detected by anti‐AtFTRc antibody (Wang *et al*., 2014), and the AtActin protein (AT3G46520) was used as an internal control and detected by anti‐actin antibody (Abclone, Wuhan, China). The histochemical staining of β‐glucuronidase (GUS) enzyme activity was performed as described by Hewezi *et al*. (2015).

### Determination of the H_2_O_2_ content in Arabidopsis

The H_2_O_2_ content was determined following a previously described method (Patterson *et al*., 1984). The OD_415_ was measured using a spectrophotometer. The H_2_O_2_ content of different samples was obtained according to the calibration curve established using various amounts of H_2_O_2_ (Fig. S4).

### MjTTL5 and AtFTRc activity assays

In order to analyze the activity of MjTTL5 and AtFTRc, the proteins were expressed by the S30 T7 High‐Yield Protein Expression System (Promega) and purified (Fig. S5); the proteins were diluted using EP buffer (0.01 M phosphate‐buffered saline (PBS), pH = 5.8, 500 mM imidazole).

The MjTTL5 activity analysis was performed as follows: 1 μl (100 ng) MjTTL5 or AtFTRc protein and PET28 protein was added to 10 μl Arabidopsis root total protein (1 μg), and then incubated at 4°C for 10 min. These mixtures were then added to 189 μl substrate solution (the substrate solution contained 100 μl 0.1% H_2_O_2_ and 89 μl distilled water), and the 1 μl EP buffer was added to 10 μl EX buffer as a control. The mixtures were then incubated at 22°C. At each time point indicated, the H_2_O_2_ content was determined as described earlier. The Arabidopsis total protein extracts were prepared as follows: 100 mg root tissues were collected and fully grounded in liquid nitrogen and then homogenized with 1 ml EX buffer (0.01 M PBS, pH = 5.8, 10 μl protease inhibitor). The homogenized materials were shaken intensely for 5–10 min before centrifugation at 20 817 ***g*** for 10 min at 4°C. The supernatants were collected and the total proteins were quantified using the Bradford method.

### PTI assay

For the determination of defense gene expression levels, 14‐d‐old seedlings were submerged in 0.01 M PBS buffer (pH 5.8) containing 10 μM flg22 (diluted in PBS buffer). After 4 h, total RNA samples were prepared from 10 mg Arabidopsis seedlings using the RNA prepmicro kit (Tiangen Biotech). The transcript abundances of WRKY33 (AT2G38470), WRKY29 (AT4G23550), CYP81F2 (AT5G57220) and FRK1 (AT2G19190) were determined by RT‐qPCR. Each sample reaction was run in triplicate. Ct values were normalized and samples were compared as described previously (Jaouannet *et al*., 2013).

Reactive oxygen species production after flg22 treatment was monitored by a luminol‐based assay on leaf disc samples. The constructs of HA and MjCBP (cellulose‐binding protein, AM491771) were used as negative controls. Leaf discs were collected and prepared for ROS assay as described by Keppler *et al*. (1989).

### *In planta* RNAi

A 350 bp fragment of the *MjTTL5* gene was amplified by PCR using the primer pair ttlFRNAiSac/ttlRRNAiXba. The fragment was digested using *Xba*I and *Sac*I, and cloned into the pTRV2 vector digested with the same enzymes for generating pTRV2:ttl5. The vectors pTRV1, pTRV2 and pTRV2:ttl5 were transformed separately into the *A. tumefaciens* EHA105. Tomato plants were infected by EHA105 carrying the corresponding constructs using procedures as previously described (Ryu *et al*., 2004). The *Tobacco rattle virus* (TRV) coat protein gene was used to verify the successful virus invasion and was detected using the primer pair TRVcpF/TRVcpR at 14 dpi (Anand *et al*., 2007).

To investigate RNAi efficiency, RNA was purified from 100 parasitic J2s (par‐J2s) collected at 5 dpi. RT‐qPCR was performed to quantify the silencing efficiency using the primer pairs qttlF/qttlR to amplify the *MjTTL5* and qactinF/qactinR to amplify the *Mj‐*β*‐actin*. Another four *TTL* isoforms from *M. javanica* were used to determine the specificity of RNAi. Independent RT‐qPCR experiments were performed three times.

### Infection assay

Thirty‐day‐old tomato plants were inoculated using 200 J2 of *M. javanica*. At 30 dpi, the roots were collected, washed and stained by acid fuchsin, and the number of females counted.

Thirty‐day‐old Arabidopsis were inoculated using 100 J2 of *M. javanica*,* M. incognita* or 100 *R. similis* nematodes per plant. At 42 dpi, the number of RKN females or *R. similis* was counted.

Each experiment was performed three times. Statistically significant differences between treatments were determined by unadjusted paired *t‐*test (*P* < 0.05) with SAS version 9.2 (SAS Institute, North Carolina, USA).

## Results

### Cloning and *in silico* analysis of the *M. javanica TTL* gene

A previous study by mass spectrometry analysis showed that *M. incognita* could secrete up to 486 proteins (Bellafiore *et al*., 2008). Among them, six proteins including a TTL protein were shown to be expressed in subventral esophageal glands by *in situ* hybridization. This TTL protein caught our attention as a further BLASTN search found that the *TTL* gene is highly conserved in various RKNs. This *TTL* gene is not the counterpart of the four reported *TTL* genes from *Radopholus similis* (Jacob *et al*., 2007). Sequence alignment analysis showed that the TTL protein at amino acid level shares a low degree of identity with the four TTL proteins from *R. similis*, including *RsTTL1* (22.2%), *RsTTL2* (18.4%), *RsTTL3* (19.6%) and *RsTTL4* (29.2%). Conversely, a BLAST search using the *TTL* gene identified by mass spectrometry analysis (Bellafiore *et al*., 2008) unveiled an unreported putative *TTL* gene in *R. similis* sharing a high sequence similarity at amino acid level (75.7%). The genes from *M. incognita* and *R. similis* were hence designated here as *MiTTL5* and *RsTTL5*, and *MjTTL5* from *M. javanica* was obtained by homology‐based PCR and characterized further in this study.

The full length of *MjTTL5* DNA is 1051 bp and the open reading frame (ORF) in cDNA is 456 bp (Fig. S6a). Southern blot analysis showed that *MjTTL5* is a single‐copy gene in the genome of *M. javanica* (Fig. S6b). The protein was predicted to contain a conserved domain, located between peptide position 35 and 109, called the domain unknown function 290 (DUF290). The protein contains a secretion signal peptide of 23 amino acids at its N‐terminal according to the signaIP program (Bendtsen *et al*., 2004), suggesting that MjTTL5 could be secreted into host cells by the nematode.

**Figure 1:**
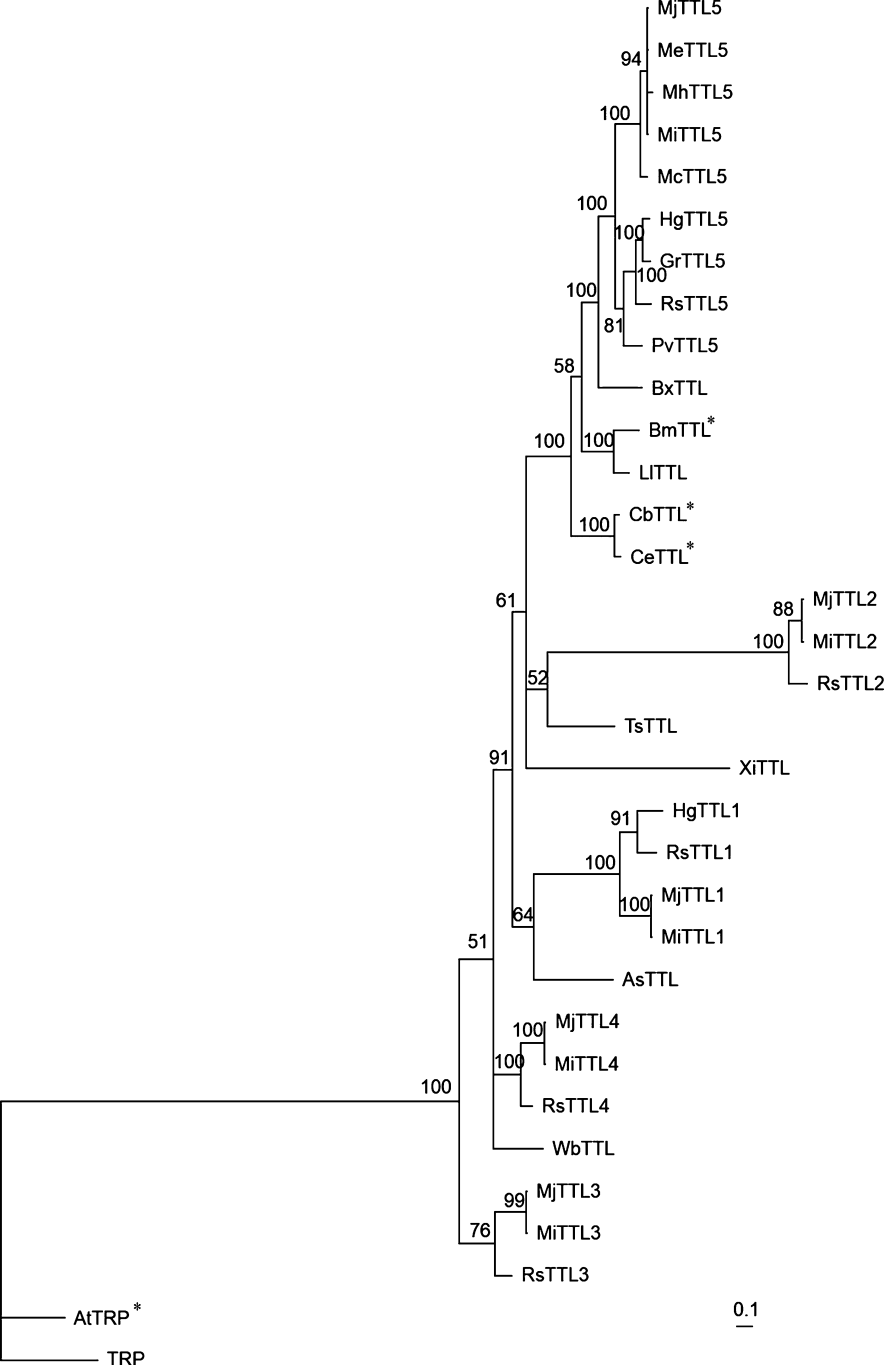
The Bayesian phylogenetic tree of transthyretin‐like protein (TTL) homologs. The posterior probability is given on each node. The tree is rooted with the transthyretin‐related protein (TRP) from *Danio rerio*. The scale bar represents branch length. The accession number of proteins used in the tree is listed in Supporting Information Table S2. *AtTRP was named as AtTTL by Nam & Li (2004); however, the amino acid sequence analysis showed that the protein contains a 5‐hydroxyisourate hydrolase domain, but not DUF290 domains. Therefore, it should be AtTRP. In addition, CbTTL, CeTTL and BmTTL were listed as CBR‐TTR‐41,TTR‐41 and BM‐TTR‐41 in the National Center for Biotechnology Information database, but the amino acid sequence analysis showed that these proteins contain a DUF290 domain, but not a 5‐hydroxyisourate hydrolase domain. Therefore we renamed them as CbTTL, CeTTL and BmTTL, respectively. Protein sequences were from *Meloidogyne javanica* MjTTL1‐MjTTL5, *Meloidogyne incognita* MiTTL1‐MiTTL5, *Meloidogyne enterolobii* MeTTL5, *Meloidogyne hapla* MhTTL5, *Meloidogyne chitwoodi* McTTL5, *Radopholus similis* RsTTL1‐RsTTL5, *Pratylenchus vulnus* PvTTL5, *Heterodera glycines* HgTTL5, *Globodera rostochiensis* GrTTL5, *Bursaphelenchus xylophilus* BxTTL,* Xiphinema index* XiTTL,* Loa loa* LlTTL,* Brugia malayi* BmTTL,* Ascaris suum* AsTTL,* Trichinella spiralis* TsTTL,* Wuchereria bancrofti* WbTTL,* Caenorhabditis briggsae* CbTTL,* Caenorhabditis elegans* CeTTL, and *Arabidopsis thaliana* AtTRP.

Sequence alignment analysis showed that MjTTL5 at the amino acid level shares a high degree of identity with putative TTL5 homologs from PPNs, including *M. incognita* (98.7%), *Meloidogyne enterlolbii* (98.7%), *Meloidogyne hapla* (96.0%), *Meloidogyne chitwoodi* (91.8%), *G. rostochiensis* (77.5%), *Pratylenchus vulnus* (76.8%), *Heterodera glycines* (75.0%), and *R. similis* (75.0%). By contrast, the amino acid sequence similarities of MjTTL5 with TTL1‐TTL4 from *M. javanica*,* M. incognita* and *R. similis* are < 44.6%. The MjTTL5 homologs can also be found in animal‐parasitic nematodes and free‐living nematodes; the highest matches are LlTTL of *Loa loa* (62.9%) and CeTTL of *Caenorhabditis elegans* (57.6%). All TTL proteins found in nematodes contain a DUF290. A phylogenetic tree (Fig. 1) was constructed to examine the relationships among 32 TTL homologs using the BI method. The BI tree shows that five TTL5 sequences from five *Meloidogyne* species, including MjTTL5, form a monophyletic clade with high support (1.00 Bayesian posterior probability (BPP)); this clade is then sister to the other clade comprising four TTL5 sequences from *H. glycines*,* G. rostochiensis*,* R. similis* and *P. vulnus*, with strong support (1.00 BPP). In addition, this phylogenetic analysis revealed that TTL1, TTL2, TTL3 and TTL4 form separate monophyly far from the TTL5 clade (Fig. 1).

### MjTTL5 is expressed in the subventral oesophageal glands and up‐regulated in the early parasitic stage of *M. javanica*

**Figure 2:**
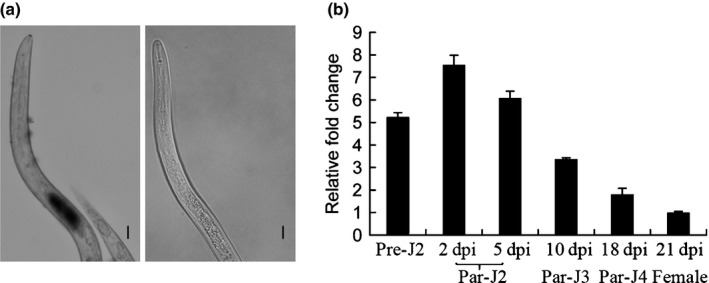
Expression patterns of MjTTL5 in *Meloidogyne javanica*. (a) Immunolocalization of MjTTL5 in a preparasitic second‐stage juvenile nematode (pre‐J2) incubated with anti‐MjTTL5 serum, showing the protein located in subventral glands (left panel), and a pre‐J2 incubated with preimmune serum showing no signal (right panel). Bars, 10 μm. (b) Developmental expression pattern of *MjTTL5*. The relative expression level of *MjTTL5* was quantified using quantitative reverse transcription PCR (RT‐qPCR) in six different *M. javanica* stages. The *β‐actin* gene was used as an internal control, and fold‐change values were calculated using the $2^{-\Delta \Delta C_{T}}$ method and relative to the expression of female stage. Data shown are the means of three repeats plus standard deviation (SD), and three independent experiments were performed with similar results. Par‐J2, Par‐J3 and Par‐J4, parasitic second‐, third‐ and fourth‐stage juveniles, respectively.

A pre‐experiment confirmed that the anti‐MjTTL5‐serum could recognize MjTTL5 specifically (Fig. S7). Immunolocalization analysis showed a strong signal within the subventral gland cells of the pre‐J2 (Fig. 2a). As expected, no signal was observed in pre‐J2s incubated with the preimmune serum (Fig. 2a).

Transcriptional expression of *MjTTL5* was analyzed by RT‐qPCR at different developmental stages of *M. javanica*. The results showed that the expression of *MjTTL5* was increased and reached a peak in the early par‐J2 at 2 dpi. Subsequently, the expression of *MjTTL5* was gradually reduced and reached a basal level at the female stage (Fig. 2b). These findings suggest that MjTTL5 plays a role in the early stages of nematode parasitism.

### MjTTL5 affects *M. javanica* parasitism

**Figure 3:**
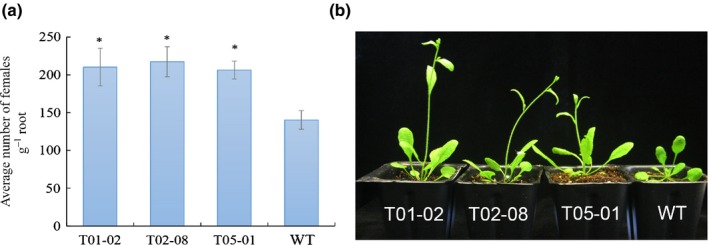
Expression of MjTTL5 in Arabidopsis enhances the susceptibility to *Meloidogyne javanica*. (a) Transgenic Arabidopsis expressing *MjTTL5* showed enhanced susceptibility to *M. javanica*. (b) Photograph of representative transgenic and wild‐type (WT) plants. Data are presented as means ± SD. The mean values significantly different from the wild‐type are denoted by an asterisk as determined by unadjusted paired *t*‐test (*P *< 0.05). The experiments were performed three times with similar results.

To investigate the role of MjTTL5 in parasitism, the transgenic Arabidopsis lines expressing MjTTL5 were generated. The expression of *MjTTL5* transcripts and MjTTL5 protein in three independent homozygous lines was confirmed by RT‐PCR and western blot (Fig. S8). The susceptibility of these transgenic Arabidopsis lines to nematode infection was then determined, and the results showed that all three transgenic lines were significantly (*P *< 0.05) more susceptible to *M. javanica* infection than wild‐type Arabidopsis, as evidenced by the statistically significant higher number of females inside the roots at 42 dpi (Fig. 3a). Similarly, the transgenic Arabidopsis lines were found to be more susceptible to *M. incognita* and *R. similis* than wild‐type Arabidopsis (Fig. S9). Intriguingly, the transgenic lines flowered earlier than wild‐type plants, with *c*. 33% of wild‐type and 57–80% of transgenic plants flowering at 25 d after sowing (Figs 3b, S10), but no difference was observed in root growth.

**Figure 4:**
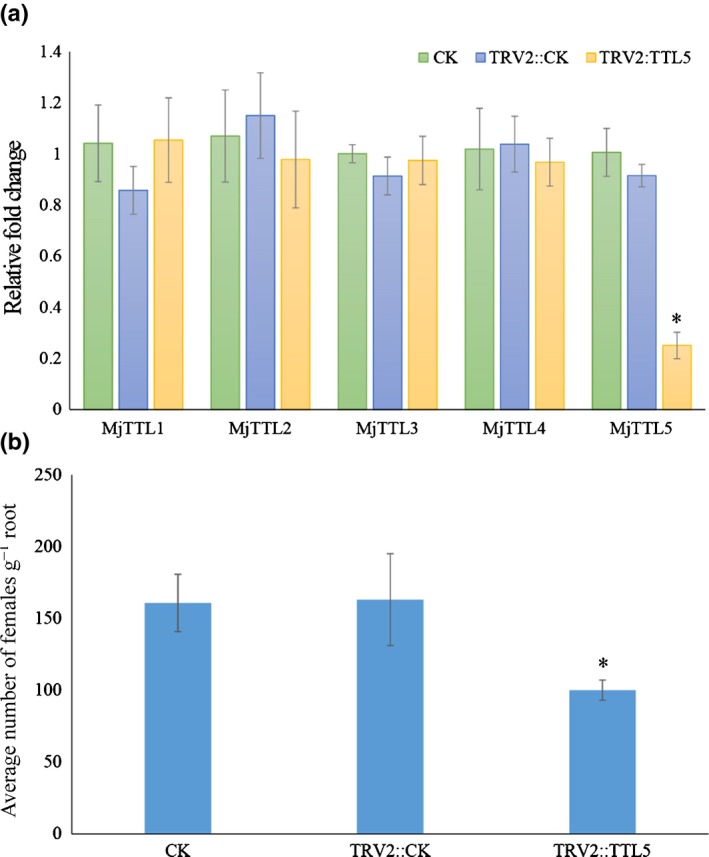
Effect of *in planta* RNAi of *MjTTL5* on *Meloidogyne javanica* parasitism. (a) Quantitative reverse transcription PCR (RT‐qPCR) assays of expression levels of *MjTTL5* in *M. javanica* collected from noninfiltrated tomato plants (CK) and pTRV2:CK, pTRV2:ttl5 agroinfiltrated plants. The expression levels of *MjTTL* isoforms from *M. javanica* were quantified to determine the specificity of RNAi. (b) The number of adult females g^–1^ root. Data are presented as means ± SD, and the mean values significantly different from the CK are denoted by an asterisk as determined by unadjusted paired *t*‐test (*P *< 0.05). The experiments were performed three times with similar results.

To further verify the role of *MjTTL5* in nematode parasitism, TRV‐mediated gene silencing was performed to knock down *MjTTL5* during parasitism of nematodes by preparing and using the RNAi construct pTRV2:ttl5. RT‐qPCR analysis showed that the transcript abundance of *MjTTL5* was drastically reduced in nematodes in the plants infiltrated with the RNAi construct compared with the control plants (Fig. 4a), demonstrating the effectiveness of *in planta* RNAi‐mediated gene silencing. Other *TTL* isoforms were used to verify the specificity of this *MjTTL5*‐targeting RNAi by RT‐qPCR analysis. The results showed that the transcriptional expression of these *TTL* isoforms were not affected by the *MjTTL5*‐targeting RNAi treatment (Fig. 4a). Significantly, the pTRV2:ttl5‐infiltrated tomato plants had *c*. 34–37.5% fewer female nematodes than noninfiltrated or the vector pTRV‐infiltrated control plants at 30 dpi (Fig. 4b). These findings again suggest that MjTTL5 plays a role in nematode parasitism.

### MjTTL5 interacts with the AtFTRc protein from host plants

**Figure 5:**
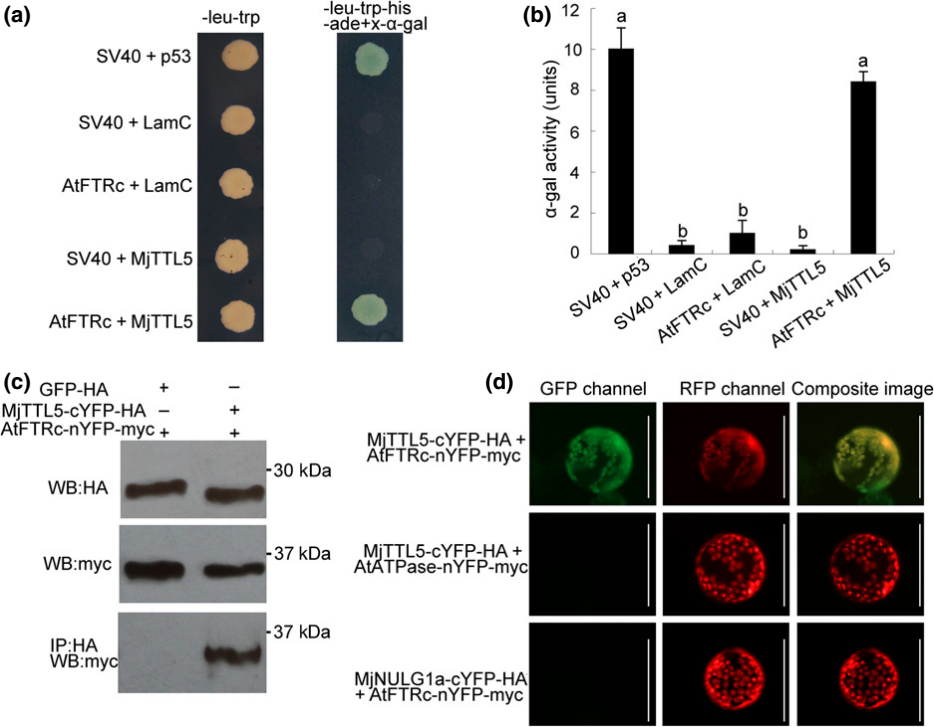
MjTTL5 interacted with AtFTRc. (a) The yeast two‐hybrid interaction between MjTTL5 and AtFTRc. Left column, cotransformants grown on SD/‐leu‐trp agar medium demonstrate that both bait and prey plasmids are present in yeast (*Saccharomyces cerevisiae*); right column, only yeast cells containing the MjTTL5 bait plus the AtFTRc prey or the positive control interaction of SV40 plus p53 grew and turned blue on the selective medium SD/‐leu‐trp‐his‐ade+x‐α‐gal agar medium. MjTTL5, p53 and LamC were cloned into the pGBKT7 vector, while AtFTRc and SV40 were cloned into the pGADT7 vector, respectively. Sv40 was cotransformed with p53 as a positive control. Sv40/LamC, ftr/LamC and sv40/MjTTL5 cotransformations were negative controls. (b) X‐α‐gal quantitative assay of the MjTTL5‐AtFTRc interaction. Five independent colonies were picked to quantify the interaction using x‐α‐gal activity. The bar represents the means of five independent colonies + SD. The columns marked with different letters are significantly different from each other as determined by Duncan's multiple range test (*P *< 0.05). The experiment was performed three times with similar results. (c) Coimmunoprecipitation analysis of AtFTRc‐nYFP‐Myc interacting with MjTTL5‐cYFP‐HA. Western blot (WB) analysis confirmed the expression of input proteins: GFP‐HA and MjTTL5‐cYFP‐HA (panel 1, anti‐HA‐antibody), AtFTRc‐nYFP‐Myc (panel 2, anti‐myc‐antibody); AtFTRc‐nYFP‐Myc was detected only after coimmunoprecipitation with the sample expressing MjTTL5‐cYFP‐HA but not the sample expressing GFP‐HA (panel 3, anti‐myc‐antibody). (d) Bimolecular fluorescent complementation visualization of the MjTTL5‐AtFTRc interaction. Tomato (*Solanum lycopersicum*) root protoplasts were cotransformed with AtFTRc‐nYFP‐Myc/MjTTL5‐cYFP‐HA, AtATP‐nYFP‐Myc/MjTTL5‐cYFP‐HA and AtFTRc‐nYFP‐Myc/NULG1a‐cYFP‐HA; the fluorescence signal was detected in the plastids of cells that were cotransformed with AtFTRc‐nYFP‐Myc/MjTTL5‐cYFP‐HA. Signals that colocalized with the plasids marker AtNTRc‐mCherry were observed in the plastids. The images were taken 24 h after cotransformation. GFP, green fluorescent protein; YFP, yellow fluorescent protein; HA, hemagglutinin. Bars, 50 μm.

To identify host proteins interacting with MjTTL5, we performed a Y2H screen using the MjTTL5 protein as a bait and a prey library prepared from the *M. javanica*‐infected Arabidopsis roots at 15 dpi. After screening, eight independent clones were identified on high‐stringency selection medium (Table S3). The interaction between MjTTL5 and these candidate receptors was further examined by cotransformation (Fig. S11), and only one protein (AtFTRc, At2g04700) was found interacting specifically with MjTTL5 (Fig. 5a). The positive interaction between AtFTRc and MjTTL5 was further confirmed by α‐galactosidase assay (Fig. 5b). AtFTRc possesses a typical active center of the ferredoxin : thioredoxin reductase, which is the key enzyme in the ferredoxin/thioredoxin system (Staples *et al*., 1996). According to sequence alignment analysis, AtFTRc is highly conserved in plants and prokaryotes (Fig. S12).

To provide solid evidence of this interaction, CoIP and BiFC assays were performed. For CoIP, the expression constructs MjTTL5‐cYFP‐HA and AtFTRc‐nYFP‐myc were coexpressed. As a negative control, the constructs GFP‐HA and AtFTRc‐nYFP‐myc were coinfiltrated into tobacco leaves. The total proteins were extracted at 48 h postinfiltration and then separated by SDS–PAGE. Upon detection by using anti‐HA antibody in immunoblot analysis, the total protein samples infiltrated with GFP‐HA or MjTTL5‐cYFP‐HA showed only one hybridization band at 28 or 27 kDa, which are the expected protein sizes of GFP‐HA and MjTTL5‐cYFP‐HA, respectively (Fig. 5c, panel 1). Similarly, detection using an anti‐myc antibody against the total protein samples treated with AtFTRc‐nYFP‐myc unveiled one band at 35 kDa (Fig. 5c, panel 2), which is the correct size of AtFTRc‐nYFP‐myc. Analysis of the immunoprecipitated protein samples showed that under the same conditions, AtFTRc‐nYFP‐myc was specifically pulled down by MjTTL5‐cYFP‐HA (Fig. 5c, panel 3), but not by GFP‐HA (Fig. 5c, panel 3).

Having different proteins in the same cellular compartments is a requirement for their interaction. A specific plastid marker, AtNTRc (Kirchsteiger *et al*., 2012), was used in this study. Fluorescence microscopy of the transformation protoplasts revealed that the GFP signal of MjTTL5 or AtFTRc is coincident with the RFP signal of AtNTRc (Fig. S13), indicating that MjTTL5 and AtFTRc are expressed in the plastids of roots. For the BiFC assay, the expression constructs, MjTTL5‐cYFP‐HA and AtFTRc‐nYFP‐myc, were coexpressed in tomato root protoplasts. The interaction between MjTTL5 and AtFTRc reconstituted the activity YFP in the transformed cells (Fig. 5d); the YFP signal is coincidence with the RFP signal of AtNTRc. As expected, no YFP signal was detected when MjTTL5 or AtFTRc was in combination with unrelated gene (Fig. 5d). The CoIP and BiFC results agree well with these Y2H findings and validate the interaction between MjTTL5 and AtFTRc *in planta*.

To test if the interaction between TTL5 and AtFTRc is specific, other *TTL* genes from different nematodes were cloned into the bait vector pGBK and cotransformed with the AtFTRc. Similarly, the homolog of AtFTRc was cloned into the prey vector pGAD and cotransformed with the MjTTL5. The cotransformation results showed that the AtFTRc could interact with MiTTL5, MeTTL5 (Fig. S14a), and RsTTL5 (Fig. S14b), but could not interact with CeTTL (Fig. S14c), MjTTL1‐MjTTL4 (Fig. S14a), and RsTTL1‐RsTTL4 (Fig. S14b). Conversely, MjTTL5 could not interact with the Arabidopsis ferredoxin: thioredoxin reductase variable subunit (AtFTRv) (Fig. S14c). These results confirmed the specific interaction between TTL5 and AtFTRc.

### AtFTRc was expressed in all tissues and its expression was induced by *M. javanica* infection

**Figure 6:**
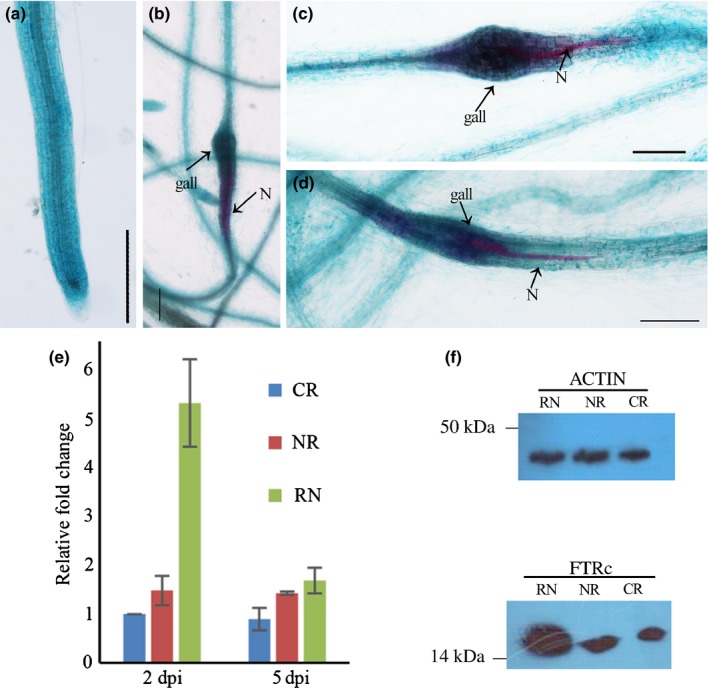
AtFTRc is up‐regulated in response to *Meloidogyne javanica* infection. (a) β‐Glucuronidase (GUS) staining in ProAtFTRc: GUS transgenic Arabidopsis roots. (b–d) GUS staining in ProAtFTRc: GUS transgenic Arabidopsis roots during *M. javanica* infection; at 2 d postinfection (dpi), AtFTRc expression was induced at the feeding site; the nematode was stained by acid fuchsine; N, nematode; bars, 200 μm. (e) AtFTRc transcripts were up‐regulated in response to *M. javanica* infection. Total RNA samples were prepared from uninfected control roots, uninfected root tissues and galls after nematode infection as described. The AtActin gene (AT1G49240) was used as an internal control and the relative fold change was relative to the expression of uninfected control roots; data are presented as means ± SD. (f) Western blot analysis showed that AtFTRc was induced in galls at 2 d post‐nematode inoculation. AtActin (AT3G46520) was used as an internal control. CR, uninfected control roots; NR, nongall zones of roots harvested from nematode infected roots; RN, gall zones of roots harvested from nematode‐infected roots.

The FTRc was initially believed to function in redox regulation in the photosystem with its expression in leaves (Staples *et al*., 1996). Recently, Balmer *et al*. (2006) reported that TaFTRc from *Triticum aestivum* was also expressed in nonphotosynthetic tissues. To confirm AtFTRc expression in the Arabidopsis roots, RT‐qPCR was performed to investigate the expression patterns of AtFTRc in different tissues. The results showed that *AtFTRc* transcripts were detected in all the examined tissues, including the roots (Fig. S15). The GUS staining also indicated that the AtFTRc was expressed in the roots (Fig. 6a). Furthermore, we found that AtFTRc expression was induced in the nematode feeding site through ProAtFtrc : GUS staining (Fig. 6b–d). Consistent with these results, RT‐qPCR and western blot analysis showed that expression of *AtFTRc* was higher in galls than in healthy roots or in uninfected tissues at 2 dpi (Fig. 6e,f). The induced expression of AtFTRc in galls indicates that AtFTRc is available at the infection site to interact with MjTTL5 secreted by *M. javanica*.

### MjTTL5 suppresses host immune responses

**Figure 7:**
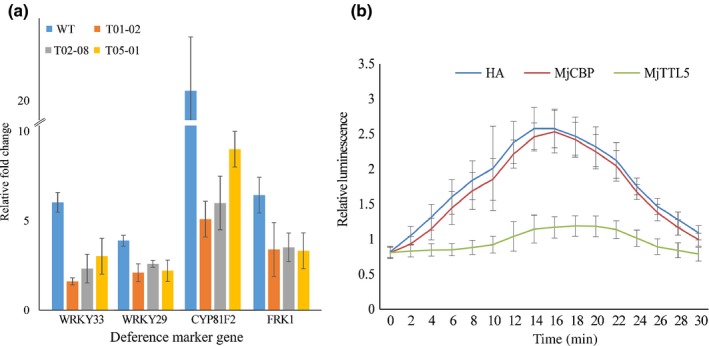
MjTTL5 suppresses the pathogen‐associated molecular pattern (PAMP)‐triggered immunity (PTI) response triggered by flg22. (a) MjTTL5 suppresses the expression of defense genes in Arabidopsis. Wild‐type and transgenic plants were grown for 14 d on half Murashige & Skoog agar medium and treated with 10 μM flg22 for 1 h. Induction of the defense marker genes WRKY33, WRKY29, CYP81F2 and FRK1 in response to flg22 treatment was determined by quantitative reverse transcription PCR (RT‐qPCR) relative to plants without treatment with flg22. Values represent the means ± SD of five plants, and the mean values significantly different from the wild‐type are denoted by an asterisk as determined by unadjusted paired *t*‐test (*P *< 0.05). WT is wild‐type Arabidopsis; T01‐02, T02‐08 and T05‐01 are different independent transgenic Arabidopsis lines expressing MjTTL5. (b) MjTTL5 suppresses flg22‐mediated reactive oxygen species (ROS) production in *Nicotiana benthamiana*. *Agrobacterium tumefaciens* strain GV3101 derivatives carrying MjTTL5, MjCBP (cellulose‐binding protein) or hemagglutinin (HA) constructs were infiltrated into the leaves of 3‐wk‐old *N. benthamiana* plants. Infiltrated leaf discs were collected 48 h postagroinfiltration and assayed for ROS production as described by Keppler *et al*. (1989). The values shown are the average of relative luminescence units (RLUs) ± SD of 18–24 leaf discs. The experiment was performed three times with similar results.

Given that silencing the AtFTRc homolog in tomato leads to enhanced disease resistance against bacterial pathogens (Lim *et al*., 2010), and that expressing MjTTL5 in Arabidopsis increased plants’ susceptibility to *M. javanica* (Fig. 3a), we hypothesized that the MjTTL5–AtFTRc interaction might interfere with the host immune responses. To test the hypothesis, we performed PTI suppression assays, including the expression levels of defense marker genes and production of ROS after triggering PTI responses in wild‐type plants and the transgenic plants expressing *MjTTL5* by using the bacterial PAMP flg22 (Felix *et al*., 1999). We compared the transcript abundances of four established defense marker genes (Jaouannet *et al*., 2013), WRKY33, WRKY29, CYP81F2 and FRK1, in Arabidopsis grown under the same conditions with or without flg22 treatment, which has been widely used to induce typical PTI responses, including activation of MAPK and expression of defense‐related genes, cell wall callose deposition and ROS burst (Boller & Felix, 2009; Fabro *et al*., 2011; Park *et al*., 2012; Chen *et al*., 2013). RT‐qPCR analysis showed that flg22 treatment of wild‐type plants strongly boosted the expression of the four defense marker genes ranging from 3.8‐ to 20‐fold higher than that of the untreated control, whereas the induction levels of these defense genes were much lower in the transgenic lines expressing MjTTL5 than in the control line (Fig. 7a).

The ROS burst is a hallmark event of PTI responses. Therefore, we investigated whether MjTTL5 could suppress the ROS production induced by flg22. The *MjTTL5*‐*HA* fusion gene, together with the vector control expressing *HA* only or expressing *Mjcbp‐HA* was introduced into tobacco leaves through agroinfiltration. Two days after infiltration, leaf discs were collected and treated with flg22 using the previously described luminol‐based method. The results showed that *in planta* expression of MjTTL5 drastically reduced the flg22‐induced ROS production in comparison with the controls (Fig. 7b). The attenuated defense gene induction and decreased ROS burst in response to flg22 treatment indicate that MjTTL5 plays a role in suppression of host plant PTI.

### MjTTL5 promotes the plant ROS‐scavenging activity

**Figure 8:**
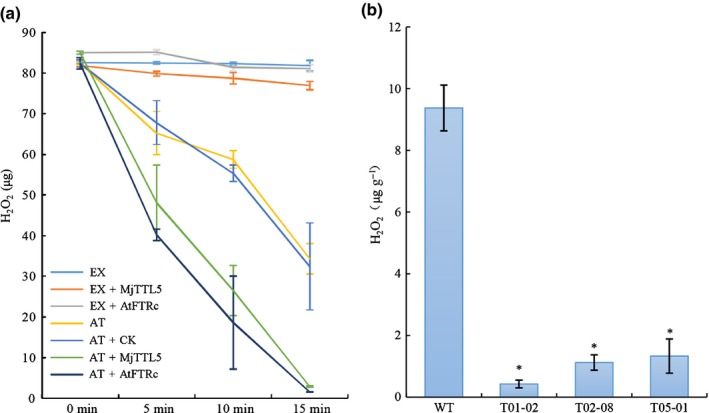
MjTTL5 alters the rate of H_2_O_2_ accumulation. (a) The peroxidase activity of Arabidopsis in the presence of MjTTL5. A total of 100 ng MjTTL5 was mixed with 1 μg Arabidopsis total proteins in the phosphate buffer containing 80 μg H_2_O_2_. The amount of H_2_O_2_ was determined at various times after the reaction, as indicated. The values are presented as means ± SD from 8 to 10 repeats. EX, Arabidopsis protein extraction buffer; TTL5, FTRc, purified MjTTL5 or AtFTRc protein; AT, Arabidopsis total protein; CK, protein samples purified from *Escherichia coli* containing empty vector pET‐28a. (b) Three independent Arabidopsis lines expressing MjTTL5 showed a significantly lower amount of H_2_O_2_ than wild‐type (WT) plants. H_2_O_2_ was quantitatively determined according to the method of Patterson *et al*. (1984). Values are presented as means ± SD. The mean values significantly different from WT are denoted by an asterisk as determined by unadjusted paired *t*‐test (*P *< 0.05). The experiments were performed three times with similar results.

Given that MjTTL5 interacts with AtFTRc, which is a key enzyme of the ferredoxin/thioredoxin system associated with the plant antioxidant defense mechanisms (Schurmann & Jacquot, 2000; Walters & Johnson, 2004; Dos Santos & Rey, 2006), we investigated whether MjTTL5 could modulate plant endogenous ROS‐scavenging activity by measuring the rate of H_2_O_2_ consumption in the reaction mixtures containing 1 μg Arabidopsis total proteins and 80 μg H_2_O_2_. In the samples containing only the plant protein extracts, H_2_O_2_ content was reduced to *c*. 35 μg at 15 min post‐reaction as a result of endogenous plant hyperoxidase activity (Fig. 8a). By contrast, when 100 ng MjTTL5 or 100 ng AtFTRc was added to this reaction mixture, the H_2_O_2_ was almost totally eliminated 15 min after the reaction (Fig. 8a). As a control, the H_2_O_2_ concentration remained almost unchanged when incubated alone with MjTTL5 or AtFTRc (Fig. 8a). Consistent with these findings, we showed that the transgenic Arabidopsis lines overexpressing MjTTL5 had significantly higher H_2_O_2_ degradation activity than the wild‐type control (Fig. 8b). Cumulatively, these results demonstrate the key role of MjTTL5 in the modulation of the plant ROS‐scavenging system.

### A portion of the DUF290 domain is required for the function of MjTTL5

**Figure 9:**
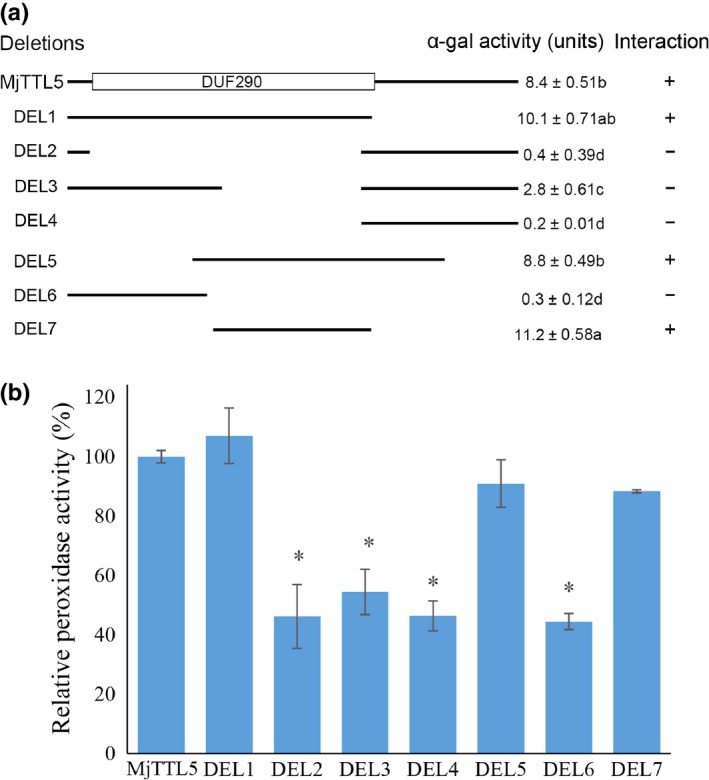
The DUF290 domain of MjTTL5 is required for its function. (a) Schematic representation of intact and truncated MjTTL5 sequences used as a bait in x‐α‐gal quantitative assay for interaction with AtFTRc in yeast (*Saccharomyces cerevisiae*). The assays were repeated twice with identical results. Symbols: −, no interaction; +, interaction; different letters indicate means that differ significantly (*P *< 0.05). (b) Relative peroxidase activity (%) of MjTTL5 and its truncated derivatives. The values significantly different from MjTTL5 are denoted by an asterisk as determined by unadjusted paired *t*‐test (*P *< 0.05). Error bars, ± SD. The experiments were performed three times and similar results were obtained. DEL1–DEL7, truncated MjTTL5 sequences.

MjTTL5 contains a domain of unknown function (DUF290) at the N‐terminal region of the peptide from the 35th to the 109th amino acid. To determine whether this domain is involved in the interaction with AtFTRc, we prepared a range of truncated derivatives of MjTTL5 for Y2H analysis. Quantitative analysis of the α‐galactosidase activity showed that three MjTTL5 derivatives, that is, Del1, Del5 and Del7, displayed similar activities to the wild‐type MjTTL5 (Fig. 9a). Among them, Del7 contains only 48 amino acids, corresponding to the MjTTL5 sequence from the 63^rd^ to the 110th amino acid, which covers a portion of the DUF290 domain (Fig. 9a). Consistent with the findings of the Y2H analysis, an *in vitro* peroxidase assay showed that, similar to MjTTL5, exogenous addition of the purified Del1, Del5 and Del7 to the total plant protein extracts could substantially boost the endogenous peroxidase activity of Arabidopsis, whereas addition of other truncated derivatives lacking or partially lacking the 63^rd^ to 110^th^ amino acid region of MjTTL5 had no effect on the plant peroxidase activity (Fig. 9b). These results unveil the key region in MjTTL5 that interacts with AtFTRc and the requirement of this interaction that modulates the plant ROS‐scavenging system.

### The *AtFTRc* mutant of Arabidopsis shows increased nematode resistance

Considering that MjTTL5 suppresses plant defense responses through interaction with AtFTRc, it is interesting to determine the role of AtFTRc in *M. javanica* parasitism. The T‐DNA insertion mutant (GK‐686B09) of At2g04700, which encodes *AtFTRc*, was obtained from TAIR. The mutant (ecotype Col‐0) contains an insertion in intron 4 of At2g04700, 941 bp downstream of the ATG initiation codon (Fig. S16a). The RT‐qPCR analysis showed that *AtFTRc* transcripts in *AtFTRc* mutants were substantially lower than that in wild‐type control (Fig. S16b).

Our previous results showed that MjTTL5 increased the ROS‐scavenging activity in wild‐type Arabidopsis (Fig. 8). If the function of MjTTL5 relies on its interaction with AtFTRc, the activity of MjTTL5 would be diminished in the *AtFTRc* mutant. We therefore compared the H_2_O_2_ reduction rates in the Arabidopsis wild‐type and *AtFTRc* mutant with or without MjTTL5. As expected, the results showed that MjTTL5 enhanced the H_2_O_2_ consumption to 2.5‐fold in wild‐type plants compared with the MjTTL5‐free treatment, whereas MjTTL5 only enhanced the H_2_O_2_ consumption 1.8‐folds AtFTRc mutants compared with the MjTTL5‐free treatment (Fig. S16c).

**Figure 10:**
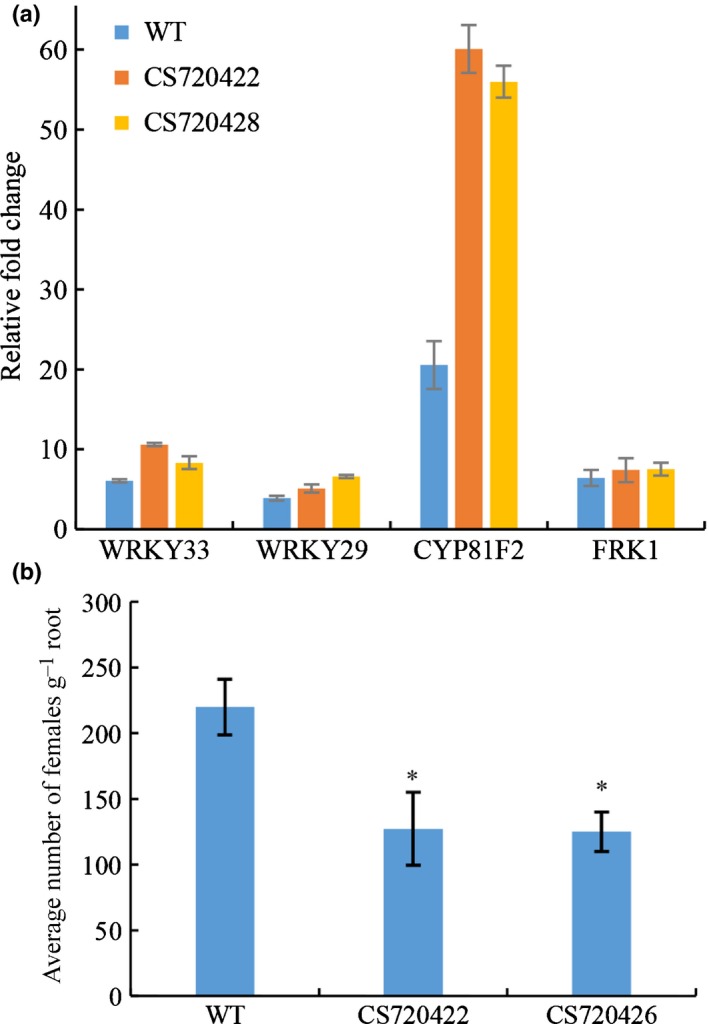
Mutation of AtFTRc affects Arabidopsis resistance to *Meloidogyne javanica*. (a) The AtFTRc mutant lines show enhanced expression of disease resistance genes after treatment with 10 μM flg22. Wild‐type (WT) plants and mutant lines were treated with 10 μM flg22. Induction of the defense marker genes FRK1, WRKY33, CYP81F2 and WRKY29 in response to flg22 was determined by quantitative reverse transcription PCR (RT‐qPCR) and presented as relative fold changes to the plants grown without flg22 treatment. The data are presented as the means ± SD of five plants. (b) The mutant lines were more resistant to *M. javanica* than the WT, as indicated by the number of females in plant roots. Values are presented as means ± SD. The mean values significantly different from the WT are denoted by an asterisk, as determined by unadjusted paired *t*‐test (*P *< 0.05). The experiments were performed three times with similar results.

Consistent with this result, the defense marker genes, especially WRKY33 and CYP81F2, were more highly expressed in the *AtFTRc* mutant lines than in the wild‐type control after treatment with the PAMP elicitor flg22 (Fig. 10a). In agreement with the effect of *AtFTRc* mutation on defense gene expression, the nematode infection assay showed that mutation of *AtFTRc* led to increased resistance against *M. javanica* parasitism with substantially fewer nematodes per transgenic lines than the wild‐type control plants (Fig. 10b).

## Discussion

The PTI response is an important plant defense mechanism encountered by parasitic nematodes during the early stages of pathogen–host interactions (Goverse & Smant, 2014), and nematodes could have evolved various mechanisms to overcome the PTI barrier. Consistent with this notion, transcriptome data showed that host plants could activate expression of defense genes at an early stage after nematode infection, but expression of the defense genes were suppressed at the subsequent stages of infection (Alkharouf *et al*., 2006; Barcala *et al*., 2010). Recently, two PPN effectors, that is, the Mi‐CRT of *M. incognita* and the GrCEP12 of *G. rostochiensis*, were shown to interfere with host plant PTI (Chen *et al*., 2013; Jaouannet *et al*., 2013), but the mechanisms of PTI suppression by these effectors remain unclear. In this study, we cloned the full‐length *MjTTL5* gene from *M. javanica* based on information from a previous mass spectrometry analysis of the nematode secretome (Bellafiore *et al*., 2008), and characterized its roles in nematode parasitism. *MjTTL5* encodes a short peptide of 151 amino acids containing a conserved domain of unknown function (DUF290). Several lines of evidence indicate that MjTTL5 is a novel nematode effector that plays a role in the modulation of plant immunity and promotion of nematode infection. First, immunolocalization analysis showed that MjTTL5 was expressed in the subventral esophageal glands, which is the common origin of nematode secreted effectors (Davis *et al*., 2004). In addition, transcriptional analysis found that *MjTTL5* transcription was up‐regulated at the early parasitic stage of *M. javanica* and declined sharply at subsequent growth stages*,* suggesting a potential role in early nematode–plant interactions. Second, Arabidopsis transgenic lines expressing *MjTTL5* became substantially more susceptible to the nematode infection than wild‐type plant controls and, vice versa, *in planta* silencing of *MjTTL5* using the RNAi approach significantly increased the plant resistance to nematode parasitism. Third, expression of MjTTL5 in plants significantly suppressed the host PTI responses, including expression of defense genes and ROS production. Fourth, and most importantly, we demonstrated, using Y2H, CoIP and BiFC, that AtFTRc, the catalytic subunit of ferredoxin: thioredoxin reductase (Dos Santos & Rey, 2006; Lim *et al*., 2010), is the target protein of MjTTL5 in Arabidopsis.

Transthyretin‐like proteins constitute a widely conserved protein family, which was first described in a paper on *in silico* analysis of protein domain families in the free‐living nematode *C. elegans* (Sonnhammer & Durbin, 1997). Subsequently, expressed sequence tag and proteomic analysis identified various TTL homologs from a range of animal‐parasitic nematodes and PPNs, such as *Haemonchus contortus* (Yatsuda *et al*., 2003), *Brugia malayi* (Hewitson *et al*., 2008) and *Trichinella spiralis* (Mitreva *et al*., 2011), *H. glycines* (Gao *et al*., 2003), *Xiphinema index* (Furlanetto *et al*., 2005), *R. similis* (Jacob *et al*., 2007), *M. incognita* (Bellafiore *et al*., 2008) and *G. pallida* (Jones *et al*., 2009). But the biological functions of these TTL proteins remain obscure. Jacob *et al*. (2007) cloned four *TTL* genes, *RsTTL1* to *RsTTL4*, from *R. similis*. Of the four *TTL* genes, *RsTTL1* was expressed in the tissues surrounding the vulva and *RsTTL2* was expressed in the ventral nerve cord (Jacob *et al*., 2007), which suggest that different TTL proteins may have different functions. In this study, MjTTL5 showed low similarity with TTL1 to TTL4, but high similarity with TTL5 proteins of other PPNs. Furthermore, the BI tree showed that TTL5 from PPNs form a monophyletic clade with strong support (Fig. 1). Consistent with the BI tree analysis, Y2H assays showed that only TTL5 proteins from PPNs can interact with AtFTRc (Fig. S14). Moreover, the FTRc is highly conserved in plants (Fig. S12), and it seems likely that the TTL5 proteins from other plant nematodes may also play similar roles in plant nematode parasitism.

FTRc is the subunit of FTR, it contains a redox‐active disulfide and a [4Fe‐4S] center and is the central enzyme of the ferredoxin/thioredoxin system. FTRc was initially found in plastids linked to photosystem and functions in redox regulation. FTRc transfers electrons from ferredoxin by light to the thioredoxins (Trx) and then to the target proteins, triggering various linked processes, and FTRv protects the Fe‐S cluster against oxygen (Staples *et al*., 1998; Schurmann & Jacquot, 2000; Dos Santos & Rey, 2006; Jones & Dangl, 2006). Recently, the protein was found in nonphotosynthetic bacteria (Balsera *et al*., 2013) and in the plastid of nonphotosynthetic tissues in plants (Balmer *et al*., 2006). Consistent with the subcellular localization of the AtFTRc, we found that the MjTTL5 was localized inside plastids (Fig. S13), indicating that the MjTTL5 and AtFTRc could interact in the plastid. The BiFC assay validated the interaction between MjTTL5 and AtFTRc in plastids. Unlike the AtFTRc, MjTTL5 lacks a chloroplast transit peptide, suggesting that a noncanonical import mechanism is utilized by MjTTL5 to enter plastids. Of note, some plant–pathogen effectors have been reported to transit into plastids through a noncanonical import mechanism, such as HopI1 and HopN1 from *Pseudomonas syringae* (Jelenska *et al*., 2007; Rodriguez‐Herva *et al*., 2012) and 140k protein from *Turnip yellow mosaic virus* (Prod'homme *et al*., 2003).

In addition, we demonstrated that the AtFTRc was expressed in root tissues and its expression could be further induced by RKN infection (Fig. 6). Given its potent redox activity, it may not be surprising to find that the ferredoxin/thioredoxin system becomes part of the antioxidant defense mechanism. For example, a chloroplastic Trx protein, CDSP32, plays a vital role in protection of the photosynthetic apparatus against oxidative damage (Broin *et al*., 2002). In agreement with this notion, our results showed that Arabidopsis could reduce H_2_O_2_ to a greater extent by exogenous addition of AtFTRc (Fig. 8a).

Consistent with the role of the ferredoxin/thioredoxin system in ROS‐scavenging, mutation or alteration of its component leads to a change in host defense responses. For example, virus‐induced gene silencing of the thioredoxin or LeFTRc of tomato led to increased accumulation of H_2_O_2_ and enhanced expression of defense‐related genes, and increased resistance to the fungal pathogen *Cladosporium fulvum* and the bacterial pathogen *P. syringae* (Rivas *et al*., 2004; Lim *et al*., 2010). Similarly, our results showed that mutation of *AtFTRc* increased the Arabidopsis resistance to *M. javanica*, whereas expression of MjTTL5 in plants caused reduced defense gene expression and attenuated disease resistance.

Recently, a new class of peroxidases, known as peroxiredoxins, were reported, which use thioredoxin to reduce ROS molecules such as H_2_O_2_ (Broin *et al*., 2002; Kotze, 2003). Previous reports inferred that FTRc plays a similar role in nonphotosynthetic plastids as it does in chloroplasts (Balmer *et al*., 2006; Kirchsteiger *et al*., 2012). We therefore hypothesized that binding of MjTTL5 to AtFTRc might cause a conformational change in AtFTRc and, consequently, enhance its efficiency of transferring electrons from ferredoxin to thioredoxin, thus facilitating elimination of ROS molecules by peroxiredoxins. These findings provide a molecular basis for understanding how MjTTL5 could enhance the nematode parasitism and diminish the disease resistance of the host plant.

Identification of MjTTL5 adds a new discovery to the list of nematode effectors that target various host mechanisms to promote parasitism. Among them, a CBP from the soybean cyst nematode *H. glycines* binds to and increases the activity of Arabidopsis pectin methylesterase, which was believed to facilitate modification of host cell walls to aid nematode infection (Hewezi *et al*., 2008). Additionally, 10A06, another effector from *H. glycines*, is able to interact with and increase the activity of Arabidopsis spermidine synthase2 (SPDS2) and, consequently, increase the activity of polyamine oxidase, which may promote induction of the host cellular antioxidant machinery (Hewezi *et al*., 2010). Furthermore, the effector SPRYSEC‐19 from *G. rostochiensis* interacts with the host immune receptor CC‐NB‐LRR Rx1 and suppresses potato disease resistance (Postma *et al*., 2012). Moreover, Y2H experiments showed that the effector Mi8D05 from *M. incognita* could interact with Arabidopsis aquaporin tonoplast intrinsic protein 2 (TIP2), suggesting a role in the regulation of solute and water transport within giant cells to promote the parasitic infection (Xue *et al*., 2013). Identification of MjTTL5 and its target protein AtFTRc presents an intriguing mechanism with which nematodes could manipulate the host biological process for their own benefit. To our knowledge, this is the first study that links a pathogen effector with the host ferredoxin : thioredoxin system in the modulation of host plant innate immunity.

Interestingly, a BLAST search showed that TTL family proteins can only be found in nematodes, suggesting that the PTI suppression mechanism employed by MjTTL5 could be different from those of the bacterial and fungal effectors. Consistent with this notion, recent studies on bacterial and fungal effectors have unveiled various inhibitory mechanisms, but none appears to interact with the host ferredoxin/thioredoxin system. The fungal effector ECP6 from *C. fulvum* was shown to suppress the PTI response by binding the PAMP chitin, which is the essential component of fungal cell wall (de Jonge *et al*., 2010), and the bacterial effector AvrPtoB from *P. syringae* pv. *tomato* interferes with the PTI signaling cascades by binding the Arabidopsis receptor kinase CERK1 or BAK1 (Gimenez‐Ibanez *et al*., 2009; Cheng *et al*., 2011). The fungal effector Pep1 from *Ustilago maydis* functions as an inhibitor of plant peroxidases (Hemetsberger *et al*., 2012), whereas the Pit2 from *Ustilago maydis* inhibits the host cysteine proteases associated with defense signaling (Mueller *et al*., 2013). Identification of MjTTL5 further broadens our understanding of effector proteins with which pathogens overcome host defense mechanisms.

## Author contributions

J.L., L.‐H.Z., B.L. and K.Z. planned and designed the research. B.L., K.Z. and S.C. performed experiments. K.Z., B.L., L.H. and L.S. analyzed data. J.L., L.‐H.Z., K.Z., B.L. and X.W. wrote the manuscript.

## Supporting information

Please note: Wiley Blackwell are not responsible for the content or functionality of any supporting information supplied by the authors. Any queries (other than missing material) should be directed to the *New Phytologist* Central Office.

**Fig. S1** T‐DNA insertion mutants of *AtFTRc* were confirmed by PCR using primers mFTRF/mFTRR/T1.
**Fig. S2** Purification of recombinant MjTTL5.
**Fig. S3** The scheme of the construct used in protoplast transformation.
**Fig. S4** The calibration curve of H_2_O_2_.
**Fig. S5** Purification of recombinant AtFTRc and MjTTL5.
**Fig. S6** Sequence analysis and Southern blot analysis of *MjTTL5*.
**Fig. S7** Western blot analysis of total proteins from preparasitic second‐stage juvenile (pre‐J2) and healthy tomato roots (TOR) with anti‐MjTTL5 serum (left panel) or preimmune serum (right panel).
**Fig. S8** RT‐PCR and western blot confirmed the expression of MjTTL5 transcripts and MjTTL5 protein in transgenic Arabidopsis.
**Fig. S9** Transgenic Arabidopsis expressing *MjTTL5* showed enhanced susceptibility to *M. incognita* (a) and *R. similis* (b).
**Fig. S10** Quantification measurement of flower stalk lengths of transgenic and wild‐type control.
**Fig. S11** Scrutinizing the interaction between candidate receptor and MjTTL5 in yeast AH109.
**Fig. S12** Multiple sequence alignment of AtFTRc and homologs.
**Fig. S13** Subcellular localization of AtFTRc and MjTTL5.
**Fig. S14** Scrutinizing the interaction between AtFTRc homologs and MjTTL5 homologs.
**Fig. S15** Expression pattern of AtFTRc transcripts in multiple plant tissues.
**Fig. S16** Characterization of *AtFTRc* Arabidopis mutants.
**Table S1** Primers used in this study
**Table S2** Accession numbers of genes or proteins used in this study
**Table S3** Candidate protein interacting with MjTTL5
**Methods S1** Protoplast isolation and transformation.
**Methods S2** The generation of constructs used in protoplast transformation.
**Methods S3** The generation of transgenic tomato roots expressing AtNTRc‐mCherry.

## References

[nph13701-bib-0001] Alkharouf NW , Klink VP , Chouikha IB , Beard HS , MacDonald MH , Meyer S , Knap HT , Khan R , Matthews BF . 2006 Timecourse microarray analyses reveal global changes in gene expression of susceptible *Glycine max* (soybean) roots during infection by *Heterodera glycines* (soybean cyst nematode). Planta 224: 838–852. 16575592 10.1007/s00425-006-0270-8

[nph13701-bib-0002] Anand A , Krichevsky A , Schomack S , Lahaye T , Tzfira T , Tang YH , Citovsky V , Mysore KS . 2007 Arabidopsis VIRE2 INTERACTING PROTEIN2 is required for *Agrobacterium* T‐DNA integration in plants. Plant Cell 19: 1695–1708. 17496122 10.1105/tpc.106.042903 PMC1913729

[nph13701-bib-0003] Asai T , Tena G , Plotnikova J , Willmann MR , Chiu WL , Gomez‐Gomez L , Boller T , Ausubel FM , Sheen J . 2002 MAP kinase signalling cascade in Arabidopsis innate immunity. Nature 415: 977–983. 11875555 10.1038/415977a

[nph13701-bib-0004] Balmer Y , Vensel WH , Cai N , Manieri W , Schurmann P , Hurkman WJ , Buchanan BB . 2006 A complete ferredoxin/thioredoxin system regulates fundamental processes in amyloplasts. Proceedings of the National Academy of Sciences, USA 103: 2988–2993. 10.1073/pnas.0511040103 PMC1413819 16481623

[nph13701-bib-0005] Balsera M , Uberegui E , Susanti D , Schmitz RA , Mukhopadhyay B , Schuermann P , Buchanan BB . 2013 Ferredoxin:thioredoxin reductase (FTR) links the regulation of oxygenic photosynthesis to deeply rooted bacteria. Planta 237: 619–635. 23223880 10.1007/s00425-012-1803-y

[nph13701-bib-0006] Barcala M , Garcia A , Cabrera J , Casson S , Lindsey K , Favery B , Garcia‐Casado G , Solano R , Fenoll C , Escobar C . 2010 Early transcriptomic events in microdissected Arabidopsis nematode‐induced giant cells. Plant Journal 61: 698–712. 20003167 10.1111/j.1365-313X.2009.04098.x

[nph13701-bib-0007] Bellafiore S , Shen ZX , Rosso MN , Abad P , Shih P , Briggs SP . 2008 Direct identification of the *Meloidogyne incognita* secretome reveals proteins with host cell reprogramming potential. PLoS Pathogens 4: e1000192. 18974830 10.1371/journal.ppat.1000192 PMC2568823

[nph13701-bib-0008] Bendtsen JD , Nielsen H , von Heijne G , Brunak S . 2004 Improved prediction of signal peptides: SignalP 3.0. Journal of Molecular Biology 340: 783–795. 15223320 10.1016/j.jmb.2004.05.028

[nph13701-bib-0009] Boller T , Felix G . 2009 A renaissance of elicitors: perception of microbe‐associated molecular patterns and danger signals by pattern‐recognition receptors. Annual Review of Plant Biology 60: 379–406. 10.1146/annurev.arplant.57.032905.105346 19400727

[nph13701-bib-0010] Broin M , Cuine S , Eymery F , Rey P . 2002 The plastidic 2‐cysteine peroxiredoxin is a target for a thioredoxin involved in the protection of the photosynthetic apparatus against oxidative damage. Plant Cell 14: 1417–1432. 12084836 10.1105/tpc.001644 PMC150789

[nph13701-bib-0011] Caboni P , Ntalli NG , Aissani N , Cavoski I , Angioni A . 2012 Nematicidal activity of (E, E)‐2,4‐decadienal and (E)‐2‐decenal from *Ailanthus altissima* against *Meloidogyne javanica* . Journal of Agricultural and Food Chemistry 60: 1146–1151. 22224661 10.1021/jf2044586

[nph13701-bib-0012] Chen S , Chronis D , Wang X . 2013 The novel GrCEP12 peptide from the plant‐parasitic nematode *Globodera rostochiensis* suppresses flg22‐mediated PTI. Plant Signaling & Behavior 8: e25359. 23803745 10.4161/psb.25359 PMC4002600

[nph13701-bib-0013] Cheng W , Munkvold KR , Gao HS , Mathieu J , Schwizer S , Wang S , Yan YB , Wang JJ , Martin GB , Chai JJ . 2011 Structural analysis of *Pseudomonas syringae* AvrPtoB bound to host BAK1 reveals two similar kinase‐interacting domains in a type III effector. Cell Host & Microbe 10: 616–626. 22169508 10.1016/j.chom.2011.10.013 PMC3876282

[nph13701-bib-0014] Davis EL , Hussey RS , Baum TJ . 2004 Getting to the roots of parasitism by nematodes. Trends in Parasitology 20: 21–28. 10.1016/j.pt.2004.01.005 15036035

[nph13701-bib-0015] Davis EL , Hussey RS , Mitchum MG , Baum TJ . 2008 Parasitism proteins in nematode–plant interactions. Current Opinion in Plant Biology 11: 360–366. 18499507 10.1016/j.pbi.2008.04.003

[nph13701-bib-0016] Dos Santos CV , Rey P . 2006 Plant thioredoxins are key actors in the oxidative stress response. Trends in Plant Science 11: 329–334. 16782394 10.1016/j.tplants.2006.05.005

[nph13701-bib-0017] Fabro G , Steinbrenner J , Coates M , Ishaque N , Baxter L , Studholme DJ , Koerner E , Allen RL , Piquerez SJM , Rougon‐Cardoso A *et al* 2011 Multiple candidate effectors from the oomycete pathogen *Hyaloperonospora arabidopsidis* suppress host plant immunity. PLoS Pathogens 7: e1002348. 22072967 10.1371/journal.ppat.1002348 PMC3207932

[nph13701-bib-0018] Fallas GA , Sarah JL . 1994 Effect of storage temperature on the *in vitro* reproduction of *Radopholus similis* . Nematropica 24: 175–177.

[nph13701-bib-0019] Felix G , Duran JD , Volko S , Boller T . 1999 Plants have a sensitive perception system for the most conserved domain of bacterial flagellin. Plant Journal 18: 265–276. 10377992 10.1046/j.1365-313x.1999.00265.x

[nph13701-bib-0020] Furlanetto C , Cardle L , Brown DJF , Jones JT . 2005 Analysis of expressed sequence tags from the ectoparasitic nematode *Xiphinema index* . Nematology 7: 95–104.

[nph13701-bib-0021] Gao BL , Allen R , Maier T , Davis EL , Baum TJ , Hussey RS . 2003 The parasitome of the phytonematode *Heterodera glycines* . Molecular Plant–Microbe Interactions 16: 720–726. 12906116 10.1094/MPMI.2003.16.8.720

[nph13701-bib-0022] Gimenez‐Ibanez S , Hann DR , Ntoukakls V , Petutschnig E , Lipka V , Rathjen JP . 2009 AvrPtoB targets the LysM receptor kinase CERK1 to promote bacterial virulence on plants. Current Biology 19: 423–429. 19249211 10.1016/j.cub.2009.01.054

[nph13701-bib-0023] Goverse A , Smant G . 2014 The activation and suppression of plant innate immunity by parasitic nematodes. Annual Review of Phytopathology 52: 243–265. 10.1146/annurev-phyto-102313-050118 24906126

[nph13701-bib-0024] Haegeman A , Mantelin S , Jones JT , Gheysen G . 2012 Functional roles of effectors of plant‐parasitic nematodes. Gene 492: 19–31. 22062000 10.1016/j.gene.2011.10.040

[nph13701-bib-0025] Hemetsberger C , Herrberger C , Zechmann B , Hillmer M , Doehlemann G . 2012 The *Ustilago maydis* effector Pep1 suppresses plant immunity by inhibition of host peroxidase activity. PLoS Pathogens 8: e1002684. 22589719 10.1371/journal.ppat.1002684 PMC3349748

[nph13701-bib-0026] Hewezi T , Baum TJ . 2013 Manipulation of plant cells by cyst and root‐knot nematode effectors. Molecular Plant–Microbe Interactions 26: 9–16. 22809272 10.1094/MPMI-05-12-0106-FI

[nph13701-bib-0027] Hewezi T , Howe P , Maier TR , Hussey RS , Mitchum MG , Davis EL , Baum TJ . 2008 Cellulose binding protein from the parasitic nematode *Heterodera schachtii* interacts with *Arabidopsis* pectin methylesterase: cooperative cell wall modification during parasitism. Plant Cell 20: 3080–3093. 19001564 10.1105/tpc.108.063065 PMC2613657

[nph13701-bib-0028] Hewezi T , Howe PJ , Maier TR , Hussey RS , Mitchum MG , Davis EL , Baum TJ . 2010 Arabidopsis spermidine synthase is targeted by an effector protein of the cyst nematode *Heterodera schachtii* . Plant Physiology 152: 968–984. 19965964 10.1104/pp.109.150557 PMC2815906

[nph13701-bib-0029] Hewezi T , Juvale PS , Piya S , Maier TR , Rambani A , Rice JH , Mitchum MG , Davis EL , Hussey RS , Baum TJ . 2015 The cyst nematode effector protein 10A07 targets and recruits host posttranslational machinery to mediate its nuclear trafficking and to promote parasitism in Arabidopsis. Plant Cell 27: 891–907. 25715285 10.1105/tpc.114.135327 PMC4558665

[nph13701-bib-0030] Hewitson JR , Harcus YM , Curwenb RS , Dowle AA , Atmadja AK , Ashton PD , Wilson A , Maizels RM . 2008 The secretome of the filarial parasite, *Brugia malayi*: proteomic profile of adult excretory‐secretory products. Molecular and Biochemical Parasitology 160: 8–21. 18439691 10.1016/j.molbiopara.2008.02.007

[nph13701-bib-0031] Huang GZ , Dong RH , Allen R , Davis EL , Baum TJ , Hussey RS . 2005 Two chorismate mutase genes from the root‐knot nematode *Meloidogyne incognita* . Molecular Plant Pathology 6: 23–30. 20565635 10.1111/j.1364-3703.2004.00257.x

[nph13701-bib-0032] Jacob J , Vanholme B , Haegeman A , Gheysen G . 2007 Four transthyretin‐like genes of the migratory plant‐parasitic nematode *Radopholus similis*: members of an extensive nematode‐specific family. Gene 402: 9–19. 17765408 10.1016/j.gene.2007.07.015

[nph13701-bib-0033] Jaouannet M , Magliano M , Arguel MJ , Gourgues M , Evangelisti E , Abad P , Rosso MN . 2013 The root‐knot nematode calreticulin Mi‐CRT is a key effector in plant defense suppression. Molecular Plant–Microbe Interactions 26: 97–105. 22857385 10.1094/MPMI-05-12-0130-R

[nph13701-bib-0034] Jaubert S , Ledger TN , Laffaire JB , Piotte C , Abad P , Rosso MN . 2002 Direct identification of stylet secreted proteins from root‐knot nematodes by a proteomic approach. Molecular and Biochemical Parasitology 121: 205–211. 12034454 10.1016/s0166-6851(02)00034-8

[nph13701-bib-0035] Jelenska J , Yao N , Vinatzer BA , Wright CM , Brodsky JL , Greenberg JT . 2007 A J domain virulence effector of *Pseudomonas syringae* remodels host chloroplasts and suppresses defenses. Current Biology 17: 499–508. 17350264 10.1016/j.cub.2007.02.028 PMC1857343

[nph13701-bib-0036] Jones JDG , Dangl JL . 2006 The plant immune system. Nature 444: 323–329. 17108957 10.1038/nature05286

[nph13701-bib-0037] Jones JT , Kumar A , Pylypenko LA , Thirugnanasambandam A , Castelli L , Chapman S , Cock PJA , Grenier E , Lilley CJ , Phillips MS *et al* 2009 Identification and functional characterization of effectors in expressed sequence tags from various life cycle stages of the potato cyst nematode *Globodera pallida* . Molecular Plant Pathology 10: 815–828. 19849787 10.1111/j.1364-3703.2009.00585.x PMC6640342

[nph13701-bib-0038] de Jonge R , van Esse HP , Kombrink A , Shinya T , Desaki Y , Bours R , van der Krol S , Shibuya N , Joosten M , Thomma B . 2010 Conserved fungal LysM effector Ecp6 prevents chitin‐triggered immunity in plants. Science 329: 953–955. 20724636 10.1126/science.1190859

[nph13701-bib-0039] Keppler LD , Baker CJ , Atkinson MM . 1989 Active oxygen production during a bacteria‐Induced hypersensitive reaction in tobacco suspension cells. Phytopathogoly 79: 974–978.

[nph13701-bib-0040] Kirchsteiger K , Ferrandez J , Pascual MB , Gonzalez M , Cejudo FJ . 2012 NADPH thioredoxin reductase C is localized in plastids of photosynthetic and nonphotosynthetic tissues and is involved in lateral root formation in Arabidopsis. Plant Cell 24: 1534–1548. 22505729 10.1105/tpc.111.092304 PMC3398562

[nph13701-bib-0041] Kotze AC . 2003 Catalase induction protects *Haemonchus contortus* against hydrogen peroxide *in vitro* . International Journal for Parasitology 33: 393–400. 12705932 10.1016/s0020-7519(03)00012-2

[nph13701-bib-0042] Kyndt T , Haegeman A , Gheysen G . 2008 Evolution of GHF5 endoglucanase gene structure in plant‐parasitic nematodes: no evidence for an early domain shuffling event. BMC Evolutionary Biology 8: 305. 18980666 10.1186/1471-2148-8-305 PMC2633302

[nph13701-bib-0043] Lee MH , Lee Y , Hwang I . 2013 *In vivo* localization in Arabidopsis protoplasts and root tissue In: Running MP, ed. G protein‐coupled receptor signaling in plants: methods and protocols. Totowa, NJ, USA: Humana Press, 113–120. 10.1007/978-1-62703-532-3_12 23913041

[nph13701-bib-0044] Lim CJ , Kim WB , Lee B‐S , Lee HY , Kwon T‐H , Park JM , Kwon S‐Y . 2010 Silencing of SlFTR‐c, the catalytic subunit of ferredoxin:thioredoxin reductase, induces pathogenesis‐related genes and pathogen resistance in tomato plants. Biochemical and Biophysical Research Communications 399: 750–754. 20705057 10.1016/j.bbrc.2010.08.016

[nph13701-bib-0045] Lin B , Zhuo K , Wu P , Cui R , Zhang L‐H , Liao J . 2013 A novel effector protein, MJ‐NULG1a, targeted to giant cell nuclei plays a role in *Meloidogyne javanica* parasitism. Molecular Plant‐Microbe Interactions 26: 55–66. 22757624 10.1094/MPMI-05-12-0114-FI

[nph13701-bib-0046] Liu Y‐G , Chen Y . 2007 High‐efficiency thermal asymmetric interlaced PCR for amplification of unknown flanking sequences. BioTechniques 43: 649–656. 18072594 10.2144/000112601

[nph13701-bib-0047] Livak KJ , Schmittgen TD . 2001 Analysis of relative gene expression data using real‐time quantitative PCR and the 2^‐▵▵CT^ method. Methods (Orlando) 25: 402–408. 10.1006/meth.2001.1262 11846609

[nph13701-bib-0048] Luciano MN , da Silva PH , Chaim OM , dos Santos VLP , Franco CRC , Soares MFS , Zanata SM , Mangili OC , Gremski W , Veiga SS . 2004 Experimental evidence for a direct cytotoxicity of *Loxosceles intermedia* (brown spider) venom in renal tissue. Journal of Histochemistry & Cytochemistry 52: 455–467. 15033997 10.1177/002215540405200404

[nph13701-bib-0049] Melillo MT , Leonetti P , Bongiovanni M , Castagnone‐Sereno P , Bleve‐Zacheo T . 2006 Modulation of reactive oxygen species activities and H_2_O_2_ accumulation during compatible and incompatible tomato‐root‐knot nematode interactions. New Phytologist 170: 501–512. 16626472 10.1111/j.1469-8137.2006.01724.x

[nph13701-bib-0050] Mitchum MG , Hussey RS , Baum TJ , Wang XH , Elling AA , Wubben M , Davis EL . 2013 Nematode effector proteins: an emerging paradigm of parasitism. New Phytologist 199: 879–894. 23691972 10.1111/nph.12323

[nph13701-bib-0051] Mitreva M , Jasmer DP , Zarlenga DS , Wang Z , Abubucker S , Martin J , Taylor CM , Yin Y , Fulton L , Minx P *et al* 2011 The draft genome of the parasitic nematode *Trichinella spiralis* . Nature Genetics 43: 228–235. 21336279 10.1038/ng.769 PMC3057868

[nph13701-bib-0052] Moffett P , Farnham G , Peart J , Baulcombe DC . 2002 Interaction between domains of a plant NBS‐LRR protein in disease resistance‐related cell death. EMBO Journal 21: 4511–4519. 12198153 10.1093/emboj/cdf453 PMC126192

[nph13701-bib-0053] Mueller AN , Ziemann S , Treitschke S , Assmann D , Doehlemann G . 2013 Compatibility in the *Ustilago maydis*‐Maize interaction requires inhibition of host cysteine proteases by the fungal effector Pit2. PLoS Pathogens 9: e1003177. 23459172 10.1371/journal.ppat.1003177 PMC3573112

[nph13701-bib-0150] Nam KH , Li JM . 2004 The Arabidopsis Transthyretin‐Like protein is a potential substrate of BRASSINOSTEROID‐INSENSITIVE 1. Plant Cell 16: 2406–2417. 15319482 10.1105/tpc.104.023903 PMC520942

[nph13701-bib-0054] Park CH , Chen SB , Shirsekar G , Zhou B , Khang CH , Songkumarn P , Afzal AJ , Ning YS , Wang RY , Bellizzi M *et al* 2012 The *Magnaporthe oryzae* effector AvrPiz‐t targets the rING E3 ubiquitin ligase APIP6 to suppress pathogen‐associated molecular pattern‐triggered immunity in rice. Plant Cell 24: 4748–4762. 23204406 10.1105/tpc.112.105429 PMC3531864

[nph13701-bib-0055] Patterson BD , Macrae EA , Ferguson IB . 1984 Estimation of hydrogen‐peroxide in plant‐extracts using titanium (IV). Analytical Biochemistry 139: 487–492. 6476384 10.1016/0003-2697(84)90039-3

[nph13701-bib-0056] Postma WJ , Slootweg EJ , Rehman S , Finkers‐Tomczak A , Tytgat TOG , van Gelderen K , Lozano‐Torres JL , Roosien J , Pomp R , van Schaik C *et al* 2012 The Effector SPRYSEC‐19 of *Globodera rostochiensis* suppresses CC‐NB‐LRR‐mediated disease resistance in plants. Plant Physiology 160: 944–954. 22904163 10.1104/pp.112.200188 PMC3461567

[nph13701-bib-0057] Prod'homme D , Jakubiec A , Tournier V , Drugeon G , Jupin I . 2003 Targeting of the Turnip Yellow Mosaic Virus 66K replication protein to the chloroplast envelope is mediated by the 140K protein. Journal of Virology 77: 9124–9135. 12915529 10.1128/JVI.77.17.9124-9135.2003 PMC187420

[nph13701-bib-0058] Rivas S , Rougon‐Cardoso A , Smoker M , Schauser L , Yoshioka H , Jones JDG . 2004 CITRX thioredoxin interacts with the tomato Cf‐9 resistance protein and negatively regulates defence. EMBO Journal 23: 2156–2165. 15131698 10.1038/sj.emboj.7600224 PMC424418

[nph13701-bib-0059] Rodriguez‐Herva JJ , Gonzalez‐Melendi P , Cuartas‐Lanza R , Antunez‐Lamas M , Rio‐Alvarez I , Li Z , Lopez‐Torrejon G , Diaz I , del Pozo JC , Chakravarthy S *et al* 2012 A bacterial cysteine protease effector protein interferes with photosynthesis to suppress plant innate immune responses. Cellular Microbiology 14: 669–681. 22233353 10.1111/j.1462-5822.2012.01749.x

[nph13701-bib-0060] Ron M , Kajala K , Pauluzzi G , Wang DX , Reynoso MA , Zumstein K , Garcha J , Winte S , Masson H , Inagaki S *et al* 2014 Hairy root transformation using *Agrobacterium rhizogenes* as a tool for exploring cell type‐specific gene expression and function using tomato as a model. Plant Physiology 166: 455–469. 24868032 10.1104/pp.114.239392 PMC4213079

[nph13701-bib-0061] Ryu CM , Anand A , Kang L , Mysore KS . 2004 Agrodrench: a novel and effective agroinoculation method for virus‐induced gene silencing in roots and diverse Solanaceous species. Plant Journal 40: 322–331. 15447657 10.1111/j.1365-313X.2004.02211.x

[nph13701-bib-0062] Schurmann P , Jacquot JP . 2000 Plant thioredoxin systems revisited. Annual Review of Plant Physiology and Plant Molecular Biology 51: 371–400. 10.1146/annurev.arplant.51.1.371 15012197

[nph13701-bib-0063] Sonnhammer ELL , Durbin R . 1997 Analysis of protein domain families in *Caenorhabditis elegans* . Genomics 46: 200–216. 9417907 10.1006/geno.1997.4989

[nph13701-bib-0064] Staples CR , Ameyibor E , Fu W , Gardet‐Salvi L , Stritt‐Etter A‐L , Schuermann P , Knaff DB , Johnson MK . 1996 The function and properties of the iron‐sulfur center in spinach ferredoxin:thioredoxin reductase: a new biological role for iron‐sulfur clusters. Biochemistry 35: 11425–11434. 8784198 10.1021/bi961007p

[nph13701-bib-0065] Staples CR , Gaymard E , Stritt‐Etter AL , Telser J , Hoffman BM , Schurmann P , Knaff DB , Johnson MK . 1998 Role of the Fe4S4 cluster in mediating disulfide reduction in spinach ferredoxin:thioredoxin reductase. Biochemistry 37: 4612–4620. 9521781 10.1021/bi9729763

[nph13701-bib-0066] Torres MA . 2010 ROS in biotic interactions. Physiologia Plantarum 138: 414–429. 20002601 10.1111/j.1399-3054.2009.01326.x

[nph13701-bib-0067] Walters EM , Johnson MK . 2004 Ferredoxin: thioredoxin reductase: disulfide reduction catalyzed via novel site‐specific 4Fe‐4S cluster chemistry. Photosynthesis Research 79: 249–264. 16328791 10.1023/B:PRES.0000017195.05870.61

[nph13701-bib-0068] Wang P , Liu J , Liu B , Da Q , Feng D , Su J , Zhang Y , Wang J , Wang HB . 2014 Ferredoxin:thioredoxin reductase is required for proper chloroplast development and is involved in the regulation of plastid gene expression in *Arabidopsis thaliana* . Molecular Plant 7: 1586–1590. 24890758 10.1093/mp/ssu069

[nph13701-bib-0069] Xue B , Hamamouch N , Li C , Huang G , Hussey RS , Baum TJ , Davis EL . 2013 The 8D05 parasitism gene of *Meloidogyne incognita* is required for successful infection of host roots. Phytopathology 103: 175–181. 23294405 10.1094/PHYTO-07-12-0173-R

[nph13701-bib-0070] Yatsuda AP , Krijgsveld J , Cornelissen A , Heck AJR , de Vries E . 2003 Comprehensive analysis of the secreted proteins of the parasite *Haemonchus contortus* reveals extensive sequence variation and differential immune recognition. Journal of Biological Chemistry 278: 16941–16951. 12576473 10.1074/jbc.M212453200

[nph13701-bib-0071] Zhang X , Henriques R , Lin SS , Niu QW , Chua NH . 2006 Agrobacterium‐mediated transformation of *Arabidopsis thaliana* using the floral dip method. Nature Protocols 1: 641–646. 17406292 10.1038/nprot.2006.97

[nph13701-bib-0072] Zipfel C , Robatzek S , Navarro L , Oakeley EJ , Jones JDG , Felix G , Boller T . 2004 Bacterial disease resistance in Arabidopsis through flagellin perception. Nature 428: 764–767. 15085136 10.1038/nature02485

