# Supplementary material for: A novel nematode effector suppresses plant immunity by activating host reactive oxygen species‐scavenging system
Source: New Phytol. 2015 Oct 20;209(3):1159–73. doi: 10.1111/nph.13701 (PMC5057313; doi:10.1111/nph.13701)
Supplement: Supplementary file 1 — Fig. S1 T‐DNA insertion mutants of AtFTRc were confirmed by PCR using primers mFTRF/mFTRR/T1. Fig. S2 Purification of recombinant MjTTL5. Fig. S3 The scheme of the construct used in protoplast transformation. Fig. S4 The calibration curve of H2O2. Fig. S5 Purification of recombinant AtFTRc and MjTTL5. Fig. S6 Sequence analysis and Southern blot analysis of MjTTL5. Fig. S7 Western blot analysis of total proteins from preparasitic second‐stage juvenile (pre‐J2) and healthy tomato roots (TOR) with anti‐MjTTL5 serum (left panel) or preimmune serum (right panel). Fig. S8 RT‐PCR and western blot confirmed the expression of MjTTL5 transcripts and MjTTL5 protein in transgenic Arabidopsis. Fig. S9 Transgenic Arabidopsis expressing MjTTL5 showed enhanced susceptibility to M. incognita (a) and R. similis (b). Fig. S10 Quantification measurement of flower stalk lengths of transgenic and wild‐type control. Fig. S11 Scrutinizing the interaction between candidate receptor and MjTTL5 in yeast AH109. Fig. S12 Multiple sequence alignment of AtFTRc and homologs. Fig. S13 Subcellular localization of AtFTRc and MjTTL5. Fig. S14 Scrutinizing the interaction between AtFTRc homologs and MjTTL5 homologs. Fig. S15 Expression pattern of AtFTRc transcripts in multiple plant tissues. Fig. S16 Characterization of AtFTRc Arabidopis mutants. Table S1 Primers used in this study Table S2 Accession numbers of genes or proteins used in this study Table S3 Candidate protein interacting with MjTTL5 Methods S1 Protoplast isolation and transformation. Methods S2 The generation of constructs used in protoplast transformation. Methods S3 The generation of transgenic tomato roots expressing AtNTRc‐mCherry. [file NPH-209-1159-s001.pdf]

New Phytologist

New Phytologist **Supporting Information**

Title: A novel nematode effector suppresses plant immunity by activating host reactive oxygen species (ROS)-scavenging system

Author: Borong Lin, Kan Zhuo, Shiyan Chen, Lili Hu, Longhua Sun, Xiaohong Wang, Lian-Hui Zhang and Jinling Liao

Article acceptance date: 31 August 2015

The following supporting information is available for this article:

Table S1 Primers used in this study

Table S2 Accession numbers of genes or proteins used in this study.

Table S3 Candidate protein interacting with MjTTL5

Fig. S1 T-DNA insertion mutants of *AtFTRc* were confirmed by PCR using primers mFTRF/mFTRR/T1.

Fig. S2 Purification of recombinant MjTTL5.

Fig. S3 The scheme of the construct used in protoplast transformation.

Fig. S4 The calibration curve of H_2O_2.

Fig. S5 Purification of recombinant AtFTRc and MjTTL5.

Fig. S6 Sequence analysis and Southern blot analysis of *MjTTL5*.

Fig. S7 Western blot analysis of total proteins from preparasitic second-stage juvenile (pre-J2) and healthy tomato roots (TOR) with anti-MjTTL5 serum (left panel) or pre-immune serum (right panel).

Fig. S8 RT-PCR and western blot confirmed the expression of MjTTL5 transcripts and MjTTL5 protein in transgenic Arabidopsis.

Fig. S9 Transgenic Arabidopsis expressing *MjTTL5* showed enhanced susceptibility to *M. incognita* (A) and *R. similis* (B).

Fig. S10 Quantification measurement of flower stalk lengths of transgenic and wild type control.

Fig. S11 Scrutinizing the interaction between candidate receptor and MjTTL5 in yeast AH109.

Fig. S12 Multiple sequence alignment of AtFTRc and homologues.

Fig. S13 Subcellular localization of AtFTRc and MjTTL5.

Fig. S14 Scrutinizing the interaction between AtFTRc homologs and MjTTL5 homologs.

Fig. S15 Expression pattern of AtFTRc transcripts in multiple plant tissues.

Fig. S16 Characterization of *AtFTRc* Arabidopis mutants.

Methods S1 Protoplast isolation and transformation.

Methods S2 The generation of constructs used in Protoplast transformation.

Methods S3 The generation of transgenic tomato roots expressing AtNTRc-mCherry.

Table S1 Primers used in this study

Primer	Sequence	References
ttlestF	AAATACTTCRGCATTAAGTAATT	This study
ttlestR	TAATTTTGCGATGRCADGGAGC	
ttl3R1	CCGATCCAGACGATACTTTGGATG	
ttl3R2	GTTCTTTATGTTTGGACACGATGTTA	
ttlcdsF	ATGTCATTATTTATTAAAAATATTTTAA	
ttlnsF	ATGAGAAAACAAGGAGTAGCCAT	
ttlcdsR	TTAAAAAATACATTCTCTTTTTTCATC	
T1	ATATTGACCATCATACTCATTGC	
mFTRF	ACTCGATGAATCTTCAAGCTGTTT	
mFTRR	AACCTTCAAGCTTTGATTTGAGA	
ttlFYEcorI	*ccggaattc* ATGAGAAAACAAGGAGTAGC	
ttlRYHind	*cccaagctt* AAAAATACATTCTCTTTTTTCATCTTC	
ttlFRnaiSac	*cgagctc* ATGAGAAAACAAGGAGTAGC	
ttlRRnaiXba	*ctagtctaga* TCAAAAGTTGATTGTAAATTAATTA	
ttlFsal	*acgcgtcgac*ATGAGAAAACAAGGAGTAGC	
ttlRsph	*acatgcatgc*TTAAAAAATACATTCTCTTTTTTCATC	
ttlFBam	CGCGGATCCATGAGAAAACAAGGAGTAGC	
ttlRPst	AAAACTGCAGAAAAATACATTCTCTTTTTTCATCTTC	
ttlFNco	*catgccatggca* ATGAGAAAACAAGGAGTAGC	
ttlRSal	*acgcgtcgac*AAAAATACATTCTCTTTTTTCATCTTC	
at2gFnde	*gggaattccatatg*ATGTCCGGTGTGGCTATTCATG	
at2gRxho	*ccgctcgag*CTATTTTGTAGGCCATGTAGCTTGA	
at4gFnde	*gggaattccatatg*ATGTCTGGTGTGGTTGCTCTTG	
at4gRxho	*ccgctcgag*CTATTTCGTTGGCCATGTCGCTT	
NBecoriF	*ccggaattc*ATGGTACAGGAGGAGAAAGCTATG	
NBxhoR	*ccgctcgag*TCATGTCCACGTACAAACGTACC	
FeFecorI	*ccggaattc*ATGAATCTTCAAGCTGTTTCTTGTA	
FeRxho	*ccgctcgag*TCACATGTTAGCTGTAGTTTCTTTT	
At5g6FecorI	*ccggaattc*ATGGAGGAGCAATTCGGCGGGAGC	
At5g6Rxho	*ccgctcgag*TCATCCATTCACGTCGCTTTTTCCC	
At5g5FecorI	*ccggaattc*ATGGCTGCCCCTTTCTATGACGAGA	
At5g5Rxho	*ccgctcgag*TCAGACAAGCGTCACATTTTCAGGTC	
at4gFsal	*acgcgtcgac*ATGTCTGGTGTGGTTGCTCTTG	
at4gFnco	*catgccatgg*CTTTCGTTGGCCATGTCGCTTGTG	
qMjTTL1F	ACTTGTGAAGGCAAACCTGCC	
qMjTTL1R	CATTACAATCGTGGTAAACATTTA	
qMjTTL2F	GCAAAATATAACAGTTCGAGGT	
qMjTTL2R	CGAATTGAACCAACCTCATTCT	
qMjTTL3F	AACAAGCTGTAGCTGTTAAAGG	
qMjTTL3R	GGATCAATAGTGGTCAACTCA	
qMjTTL4F	CGTTTGCCGTTCGTACACAAT	
qMjTTL4F	TTTGTAGTTTCAACAGTTGAAC	
qAtFTRF	GAGCGAAAACGGAACCGTCG	
qAtFTRR	GTCTGCAAGGGCAAAGCGGT	
qAtACTF	CAGCTCTTGCCCCGAGCAGC	
qAtACTR	TGTGGACAATGCCTGGACCTG	
T7TTLF	GGATCCTAATACGACTCACTATAGGGGTGGAAATACTTCGGCATTAAGTA	
T7TTLR	GGATCCTAATACGACTCACTATAGGG CAAAAGTTGATTGTAAATTAATTATTC	
TTLF	GTGGAAATACTTCGGCATTAAGTA	
TTLR	CAAAAGTTGATTGTAAATTAATTATTC	
qTTLF	ATGAGAAAACAAGGAGTAGCCAT	
qTTLR	TATCAGTTAATTCTCTAGTATATCC	
MjcbpF	acgggggactctagaATGGATGATGCTGGTAGATATCCTTC	

MjcbpR	atcataagggtaggatcc TTCAATAATGTTGCATCCTTCTTCTTG	
WRKY3F	GCTGCTATTGCTGGTCACTCC	Jaouannet *et al.* (2013)
WRKY3R	GGTCTCCTCGTTTGGTTCTTCC	
WRKY2F	ATCCAACGGATCAAGAGCTG	
WRKY2R	GCGTCCGACAACAGATTCTC	
CYP8F	GTGAAAGCACTAGGCGAAGC	
CYP8R	ATCCGTTCCAGCTAGCATCA	
UBIF	GCCAAAGCTGTGGAGAAAAG	
UBIR	TGTTTAGGCGGAACGGATAC	
FRK1F	TGCAGCGCAAGGACTAGAG	
FRK1R	ATCTTCGCTTGGAGCTTCTC	
Del1R	*acgcgtcgac* GCGATGACAAGGAGCTTCCAAT	
Del2F	AAAATTAAATTTTTAATTCCTAAAAAATTT	
Del2R	AATGGCTACTCCTTGTTTTCTCAt	This study
Del3R	TTCATCCAAAGTATCGTCTGGATC	
Del4F	*catgccatggca* atgCCTTGTCATCGCAAAATTAAATTTT	
Del5F	*catgccatggca* atgAGTAATTCTACAAAAGTTAGAAT	
Del5R	*acgcgtcgac* AATTAAAAATTTAATTTTGCGATG	
Del6R	*acgcgtcgac* GTCTGGATCGGGACCTGTATCAATAT	
Del7F	*catgccatggca* atgCCAGACGATACTTTGGATGAAAAAT	
FTRcproF	gaccatgattacgccaagcttTTGAACCAAAACAAATTCTTCC	
FTRcproR	tctacaggacgtaacatACCAAGTGGACTCGAAAGGAATCCG	
NTRcF	tggagagaacacgggggacATGGCTGCGTCTCCCAAGATA	
NTRcR	tcgcccttgctcaccatggcataatcaggtacatcataagggtatctagaTTTATTGGCCTCAATGAATTC	
RFPF	ATGGTGAGCAAGGGCGAGGAG	
RFPpbiR	tgaacgatcggggaaattCTTACTTGTACAGCTCGTCCATGCC	
TTLuniF	agcccaagcttcgactctagATGAGAAAACAAGGAGTAGC	
TTLuniR	agctcctcgcccttgctcacAAAAATACATTCTCTTTTTTCATC	
FTRuniF	agctcctcgcccttgctcacAAAAATACATTCTCTTTTTTCATC	
FTRuniR	agctcctcgcccttgctcacCATGTTAGCTGTAGTTTCTTTTAT	
MiTTL5F	*catatggccatggaggcc*ATGAGAAAACAAGGAGTAGCCA	
MiTTL5R	*ggccgctgcaggtcgac*TTAAAAAATACATTCTCTTTTTTCATCTTC	
MeTTL5F	*catatggccatggaggcc*ATGAGAAAACAAGGAGTAGC	
MeTTL5R	*ggccgctgcaggtcgac*AAAAATACATTCTCTTTTTTCATCTTC	
RsTTL5F	*catatggccatggaggcc*ATGCGCAAACAGGGTGTGG	
RsTTL5R	*ggccgctgcaggtcgac*AAAGATGCACTCCCTCTTCTCTTC	
RsTTL1F	*catatggccatggaggcc*ATGGCGCCTATGTTTCTGCCTA	
RsTTL1R	*ggccgctgcaggtcgac*TCAATGAAGACAGTCACGCTCC	
RsTTL2F	*catatggccatggaggcc*GATGTGCAGAACATCACTGTGC	
RsTTL2R	*ggccgctgcaggtcgac*GTCGCATGTCTCGGTGTCGC	
RsTTL3F	*catatggccatggaggcc*ATGCGCCAACAGGCGGTCGC	
RsTTL3R	*ggccgctgcaggtcgac*TCAGAGCAAATCTCGCTCCTCG	
RsTTL4F	*catatggccatggaggcc*ATGTGTGGTGCGGCACCGGCG	
RsTTL4R	*ggccgctgcaggtcgac*CTAGAGACGAACGAAAAAGTTGAATT	
MjTTL1F	*catatggccatggaggcc*ATGACTTTAATTTTAACATT	
MjTTL1R	*ggccgctgcaggtcgac*TCAATGAATACAGTCACGTTC	
MjTTL2F	*catatggccatggaggcc*GACCAGCAAAATATAACAGTTCGAGG	
MjTTL2R	*ggccgctgcaggtcgac*TGGTATATCAATTTCTGTTATT	
MjTTL3F	*catatggccatggaggcc*ATGAGACAACAAGCTGTAGCTGT	
MjTTL3R	*ggccgctgcaggtcgac*AGAAGAACGAGACCTTCTTTGA	
MjTTL4F	*catatggccatggaggcc*ATGTTTAATATCTTGTATTCAAC	
MjTTL4R	*ggccgctgcaggtcgac*GAAAATGAACTAATTGACGATATTATTTTT	

Table S2 Accession numbers of genes or proteins used in this study

Abbreviation	Species	NCBI Accession No.
MjTTL1	*Meloidogyne javanica*	KR864894
MjTTL2	*Meloidogyne javanica*	KR912078
MjTTL3	*Meloidogyne javanica*	KR864895
MjTTL4	*Meloidogyne javanica*	KR864896
MjTTL5	*Meloidogyne javanica*	KR604797
MiTTL1	*M. incognita*	CF802755
MiTTL2	*M. incognita*	CN577982
MiTTL3	*M. incognita*	JK297947
MiTTL4	*M. incognita*	AW783163
MiTTL5	*M. incognita*	CD749147
RsTTL1	*Radopholus similis*	CAM84510
RsTTL2	*Radopholus similis*	CAM84511
RsTTL3	*Radopholus similis*	CAM84512
RsTTL4	*Radopholus similis*	CAM84513
RsTTL5	*Radopholus similis*	EY191372
MeTTL5	*M. enterolobii*	KR864898
MhTTL5	*M. hapla*	BM900463
McTTL5	*M. chitwoodi*	CB930705)
PvTTL5	*Pratylenchus vulnus*	EL889277
HgTTL1	*Heterodera glycines*	CB375824
HgTTL5	*Heterodera glycines*	CB375824
GrTTL5	*Globodera rostochiensis*	BM344869
BxTTL	*Bursaphelenchus xylophilus*	CJ981198
XiTTL2	*Xiphinema index*	CAH89267
LlTTL	*Loa loa*	XP_003143890
BmTTL*	*Brugia malayi*	CDP99567
AsTTL	*Ascaris suum*	ERG80249
TsTTL	*Trichinella spiralis*	XP_003381366
WbTTL	*Wuchereria bancrofti*	EJW79115
CbTTL*	*Caenorhabditis briggsae*	XP_002630351
CeTTL*	*C. elegans*	NP_001293509
AtTRP*	*Arabidopsis thaliana*	NP_200630
TRP	*Danio rerio*	Q06S87

*Named as AtTTL by Nam & Li (2004). However, the amino acid sequence analysis showed that the protein contain a 5-hydroxyisourate hydrolase domain, not contain DUF290 domains. Therefore, it should be transthyretin-related protein (TRP), namely AtTRP. In addition, CbTTL, CeTTL and BmTTL were listed as CBR-TTR-41, TTR-41 and BM-TTR-41 in the NCBI database, but the amino acid sequence analysis showed that these proteins contain a DUF290 domain, not contain a 5-hydroxyisourate hydrolase domain. Therefore we renamed them as CbTTL, CeTTL and BmTTL, respectively.

Table S3 Candidate protein interacting with MjTTL5

Clone [a]	Locus tag	Accession no.	Gene
1	AT5G50230	NP_199834	nucleotide binding
2	AT4G32530	NP_001119099	Arabidopsis thaliana vacuolar ATP synthase
3	AT2G04700	NM_126500	ferredoxin thioredoxin reductase catalytic subunit
4	AT4G32530	NP_001119099	Arabidopsis thaliana vacuolar ATP synthase
5	AT5G50230	NP_199834	nucleotide binding
6	At2g25610	NM_128119	Arabidopsis thaliana ATPase, F0/V0 complex, subunit C protein
7	AT5G64180	AY114022	hypothetical protein
8	AT5G55060	NM_001203612	Arabidopsis thaliana uncharacterized protein

[a]The clone 1 and clone 5 contain the sequence from 1st to 149th and from 253rd to 509th of the NP_199834; the clone 2 and clone 4 contain the sequence from 1st to 210th and from 1st to 87th of the NP_001119099. The clone 2, 4, 5, 6, 7, 8 were found once, the clone 3 was found twice with identical sequence and the clone 1 was found three times with identical sequence.

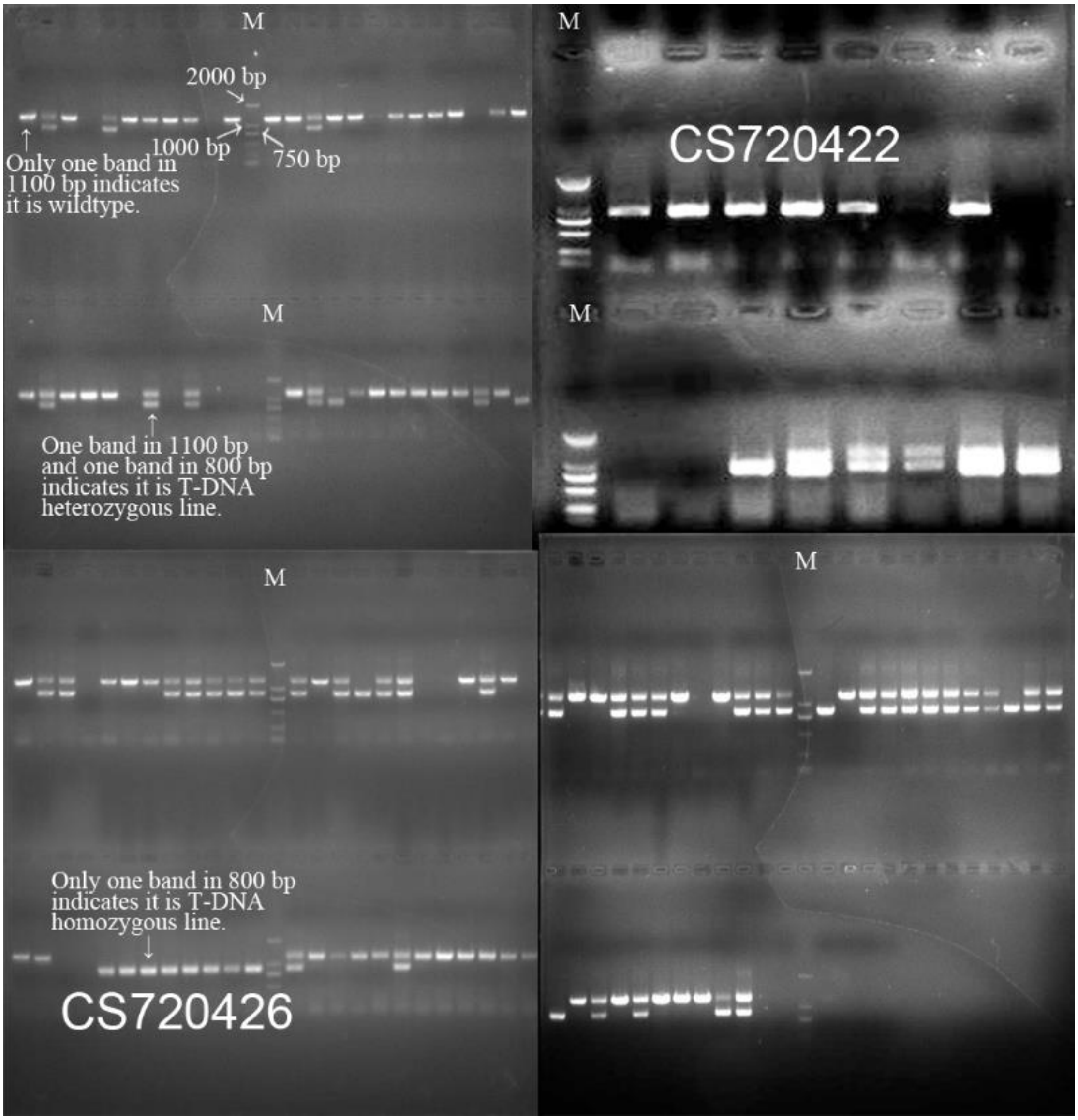

Fig. S1 T-DNA insertion mutants of *AtFTRc* were confirmed by PCR using primers mFTRF/mFTRR/T1. The primer mFTRF/mFTRR amplified the fragment between 1646956 bp and 1648158 bp of the *Arabidopsis thaliana* chromosome 2 (Accession no.: gb|CP002685.1|) showing one band of *c*. 800 bp in the homozygote T-DNA insertion lines, two bands of *c*. 800 bp and 1200 bp in the heterozygote T-DNA insertion lines and one band of *c*. 1200 bp in the wild type plants. The homozygous seed stocks (CS720422 and CS720426) were used in the study. M, DNA molecular marker Ds2000.

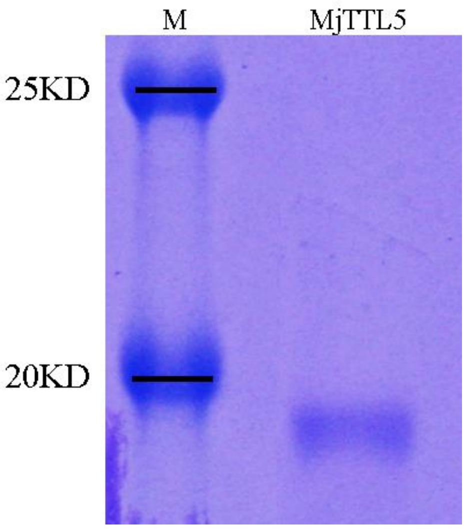

Fig. S2 Purification of recombinant MjTTL5. SDS-PAGE (15%) analysis of recombinant MjTTL5 protein stained with Coomassie brilliant blue; M, the proteins standard molecular weight.

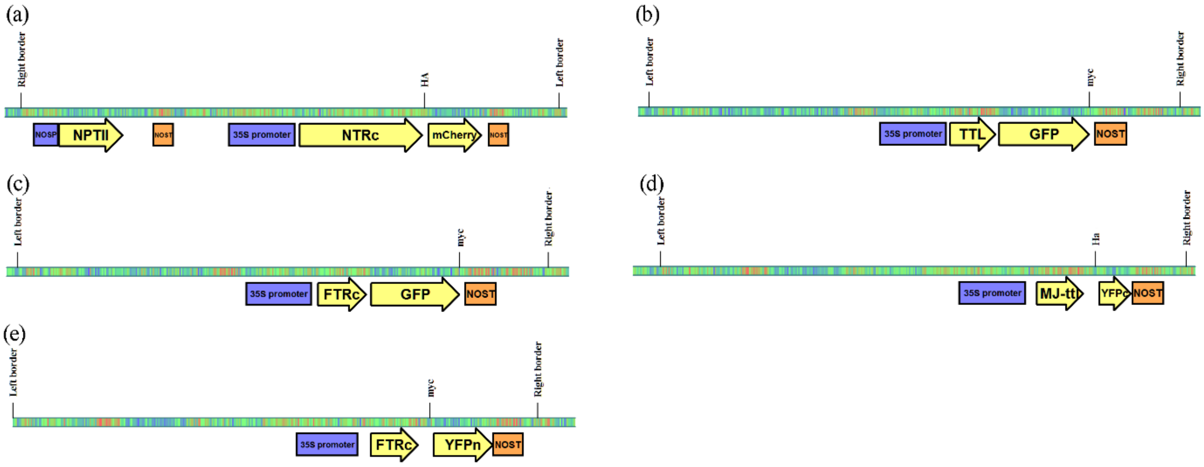

Fig. S3 The scheme of the construct used in protoplast transformation. (a) The construct used to generate the transgenic Arabidopsis expressing the plastid marker AtNTRc-mCherry; (b) , (c) The constructs used to investigate the subcellular location of MjTTL5 and AtFTRc; (d), (e) The constructs used in BiFC assay; NOSP, nos promoter, NOST; nos terminator; NPTII, neomycin phosphotransferase II gene; mCherry, GFP, red and green fluorescent protein; YFPc, YFPn, the C-terminal or N-terminal of yellow fluorescent protein.

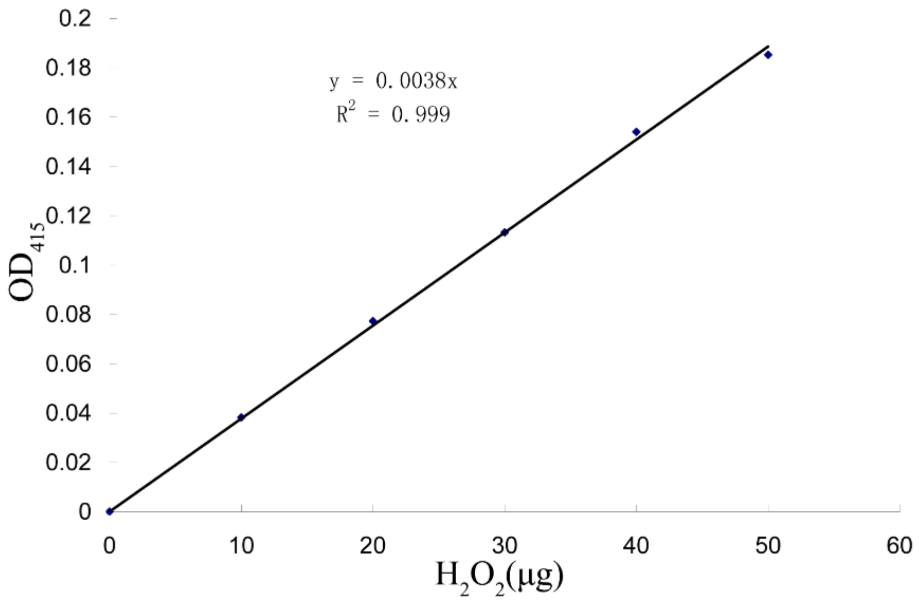

Fig. S4 The calibration curve of H_2O_2.

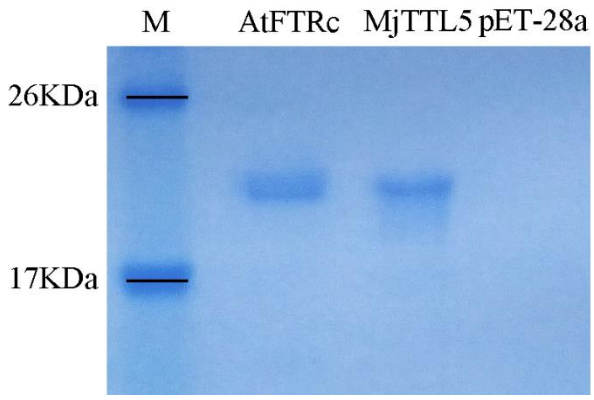

Fig. S5 Purification of recombinant AtFTRc and MjTTL5. SDS-PAGE (15%) analysis of recombinant AtFTRc and MjTTL5 protein stained with Coomassie brilliant blue; M, the protein standard molecular weight.

(a)

ATGTCATTATTTATTAAAAATATTTTAATTTTATTTACAACAATTTTAGTTTGG
Start codon
ATATTTCGATTAATTAATTTTTTAAAAAAAAGGGGGAGGGGGAGGGCTCTT
GTCTAATCATAATGGAAATTTTTATGTATCCAAACTAAGTACCCGATTTTTGT
GCTAGAATATATGATTCGGTATTCCGTGTAACTGAATTTAGGGTGAGTAGAC
ACTCATTGCCGGTTACTTAGACGGATGAAACTTTGATTGGACGTTAATCAGT
ATGCACAAGCCAATTGTTGTTTTTAAAAATTAAATTTTAATAAGTTATACATA
AATAGAAGAAATAAAATTAAAAAAATTTTTTAATCCCTTTTCCCCGTTATTTT
AACAAAAATAATTTAAATTTTTTATTAAAAAAAATTGTTTTTACAAAAATTTT
CAAAAAAAAAATATTAATAAATATCCAGTTTATTATTTTGAATAATTCCAATG
TATTTATGAGAAAACAAGGAGTAGCCATTAGCGGAAGATTTATTTGTGGAA
ATACTTCGGCATTAAGTAATTCTACAAAAGTTAGAATTGTTGATATTGATACA
GGTAATTAATTAGTTTTTTTAAATTAATTATTTTGTTTTTTAAAAAAAGGTCC
CGATCCAGACGATACTTTGGATGAAAAATTTGTTGATGCAACTGGGGCATTT
AAATTAAATGGATATACTAGAGAATTAACTGGTTTAGTTAATTAAAAAAGAA
AATATTTTTATTCTAATTTAATTTAGATATAGAcCCCGTTCTTTATGTTTGGAC
ACGATGTTATTCATTGGAAGCTGTAAATATTTTATTAAATATTCAAAAAACTC
TTTCCTATTTTTATTTTAGCCTTGTCATCGCAAAATTAAATTTTTAATTCCTAA
AAAATTTATTATCGGAGAAGAACCTAAATTAAATGAATGGCTAGATATTGGA
ATAATTAATTTACAATCAACTTTTGAAGGTTTTTTTAAAAAAATTAATTATTT
TTAAATATTTTTTTAAATTTGAAGATGAAAAAAGAGAATGTATTTTTTAA
Stop codon

(b)

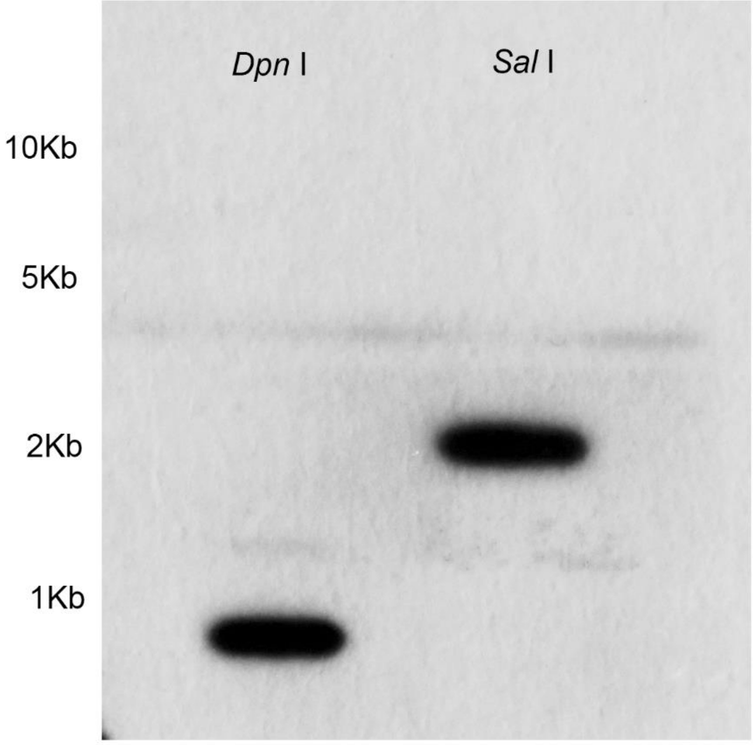

Fig. S6 Sequence analysis and Southern blot analysis of *MjTTL5*. (a) The DNA sequence of *MjTTL5*, the predicted start codon and stop condon are boxed, five introns are presented in red. (b) *MjTTL5* is a single copy gene in *M. javanica* genome. Genomic DNA of *M. javanica* was digested with *Dpn* I and *Sal* I and probed with digoxigenin labeled *MjTTL5* DNA.

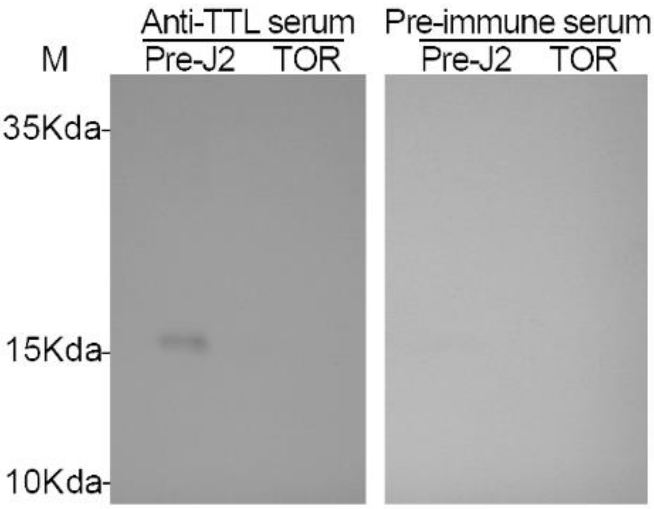

Fig. S7 Western blot analysis of total proteins from preparasitic second-stage juvenile (pre-J2) and healthy tomato roots (TOR) with anti-MjTTL5 serum (left panel) or pre-immune serum (right panel).

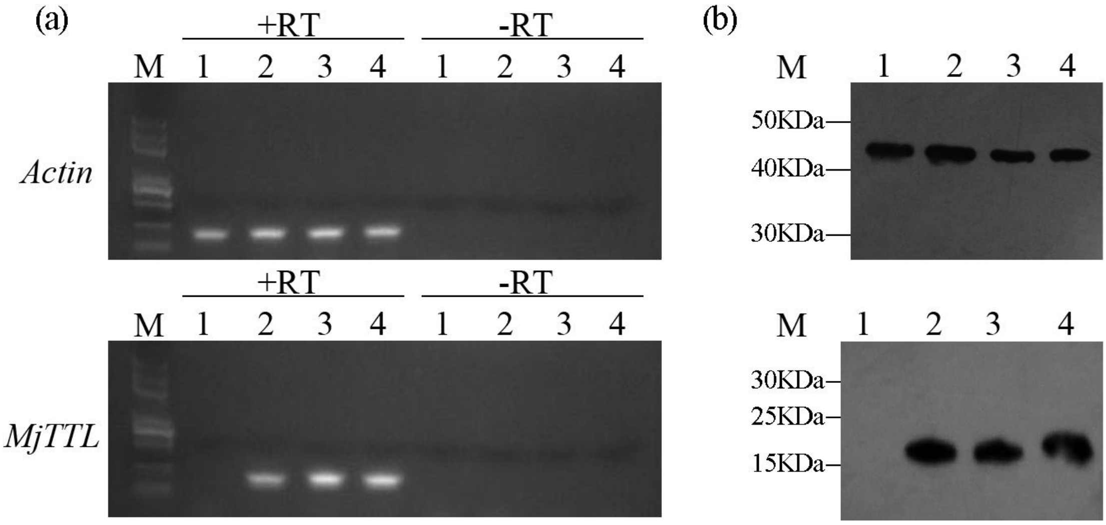

Fig. S8 RT-PCR and western blot confirmed the expression of *MjTTL5* transcripts and MjTTL5 protein in transgenic Arabidopsis. (a) RT-PCR of *MjTTL5* transcripts, the Arabidopsis actin gene as an internal control. +RT and –RT indicate the presence or absence of reverse-transcriptase, respectively, in the cDNA synthesis reaction. (b) Western blot confirmation of the MjTTL5 product with anti-MjTTL5 serum (botton panel), lane 1 is wild type Arabidopsis, lane 2 (T01-02), lane 3 (T02-08) and lane 4 (T05-01) represent different independent transgenic Arabidopsis lines expressing *MjTTL5*. The Arabidopsis actin is used as an internal control (top panel).

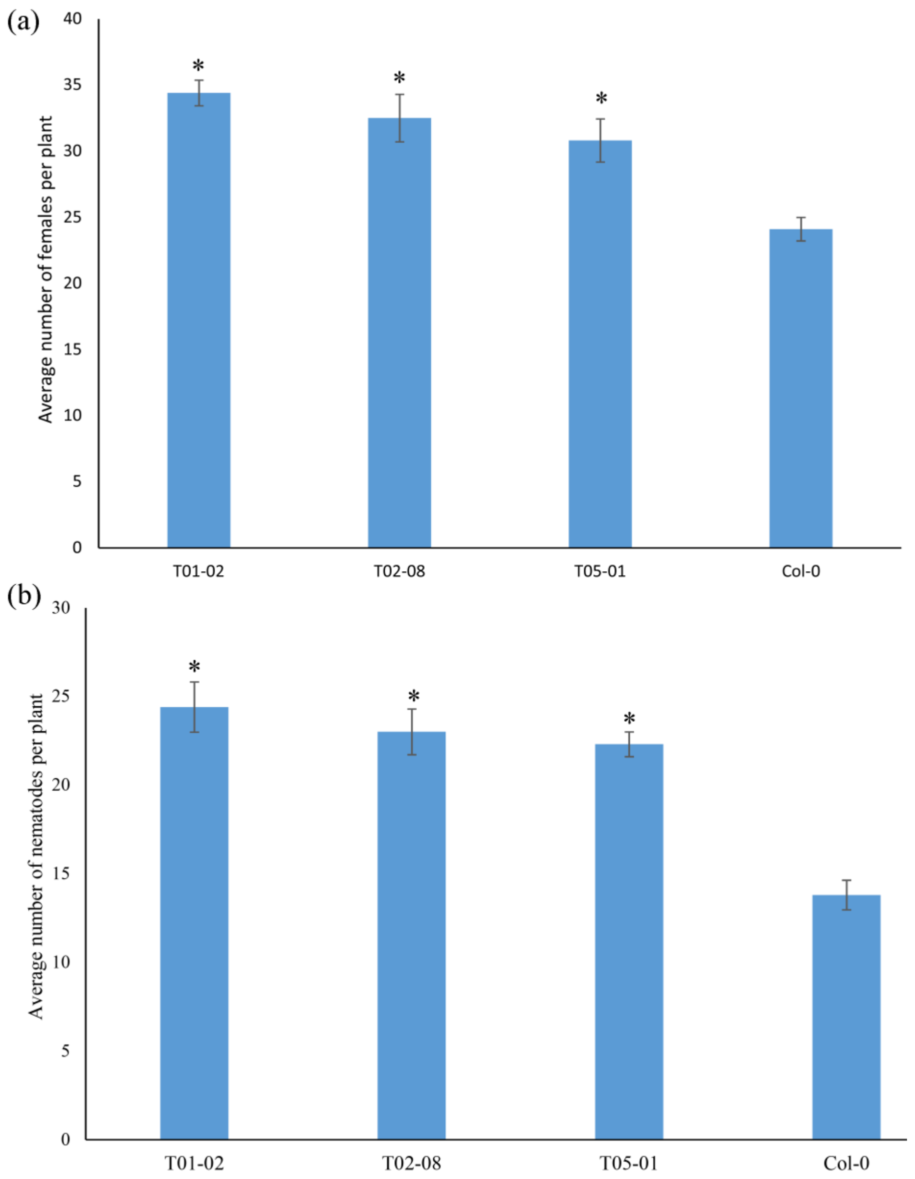

Fig. S9 Transgenic Arabidopsis expressing *MjTTL5* showed enhanced susceptibility to *M. incognita* (a) and *R. similis* (b). Data are presented as the means ± SD. The mean values significantly different from the wild type are denoted by an asterisk as determined by unadjusted paired *t* test (P<0.05). The experiments were performed three times with similar results.

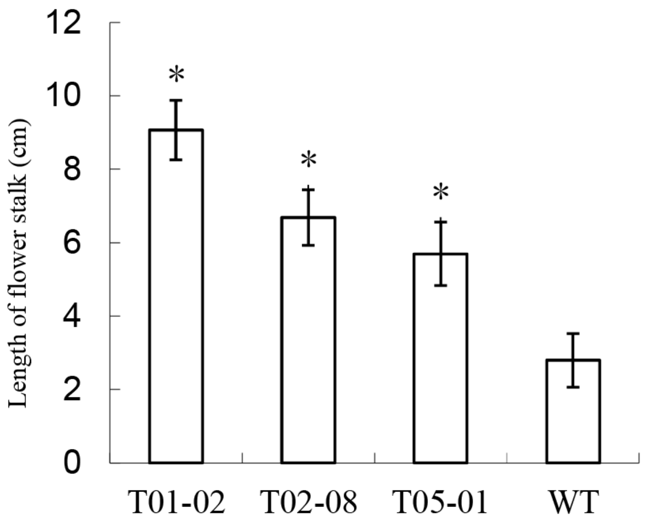

Fig. S10 Quantification measurement of flower stalk lengths of transgenic and wild type control. Data are presented as the means ± SD. The mean values significantly different from the wild type are denoted by an asterisk as determined by unadjusted paired *t* test ($P<0.05$). The experiments were performed three times with similar results.

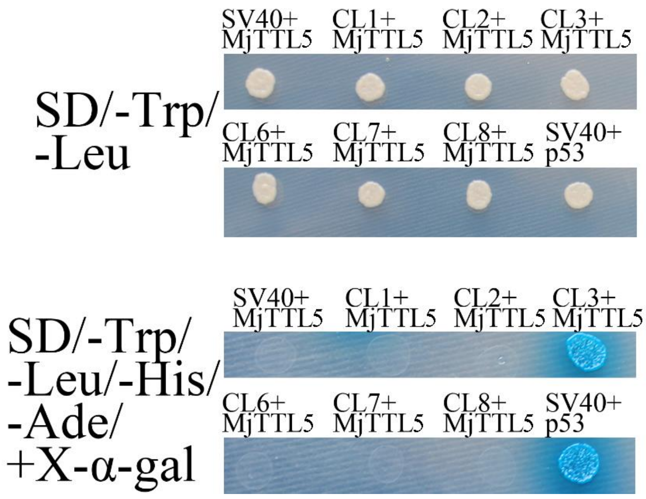

Fig. S11 Scrutinizing the interaction between candidate receptor and MjTTL5 in yeast AH109. The corresponding genes encoding the SV40 large T-antigen protein (SV40), *Arabidopsis thaliana* nucleotide binding protein (CL1), *Arabidopsis thaliana* vacuolar ATP synthase (CL2), ferredoxin thioredoxin reductase catalytic subunit (CL3), *Arabidopsis thaliana* ATPase F0/V0 complex subunit C protein (CL6), *Arabidopsis thaliana* hypothetical protein (CL7), *Arabidopsis thaliana* uncharacterized protein (CL8) and the fragment of murine p53 protein (aa 72–390) (p53), were cloned in the vector plasmid. The resultant expression constructs were co-transformed with the MjTTL5-bait construct to the yeast strain AH109. SV40+MjTTL5 and SV40+p53 combination were used as the negative control and the positive control, respectively.

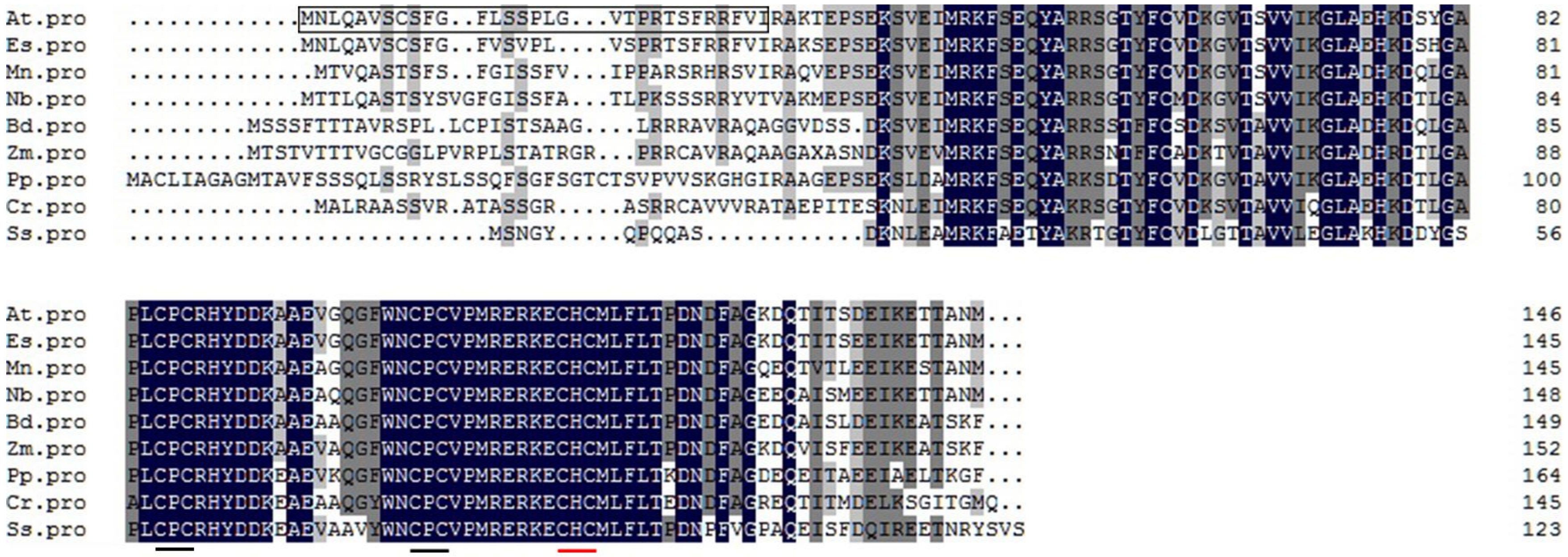

Fig. S12 Multiple sequence alignment of AtFTRc and homologues. Identical amino acid residues are marked with black, similar amino acid residues are marked dark grey and grey. Dashes indicate the absence of residues. The active site [4Fe–4S] clusters are composed of two conserved CPC and one CHC motifs, which are underlined by black line and red line, respectively. The chloroplast transit peptide was predicted by ChloroP 1.1 Server and inditated by black box. At, *Arabidopsis thaliana* (NP_178547), Es, *Eutrema salsugineum* (XP_006405543), Mn, *Morus notabilis* (EXC45070), Nb, *Nicotiana benthamiana* (AGK06902), Bd, *Brachypodium distachyon* (XP_003574776), Zm, *Zea mays* (ACG38750), Pp, *Physcomitrella patens* (XP_001754387), Cr, *Chlamydomonas reinhardtii* (XP_001693262), Ss, *Synechococcus* sp. (WP_015123106).

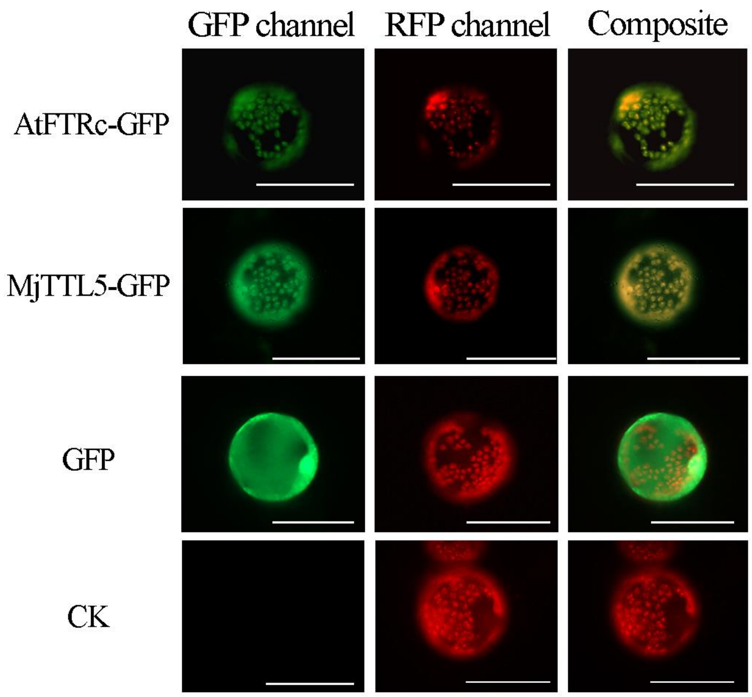

Fig. S13 Subcellular localization of AtFTRc and MjTTL5. AtFTRc and MjTTL5 cDNA were fused in frame with the coding sequence of green fluorescent protein (GFP), respectively, and expressed in tomato root protoplast. The AtNTRc is a plastid-localized protein and used as a marker to indicated the plastid. Free GFP was used as control, CK represents the protoplast was not transformed any construct. Bar, 50 µm.

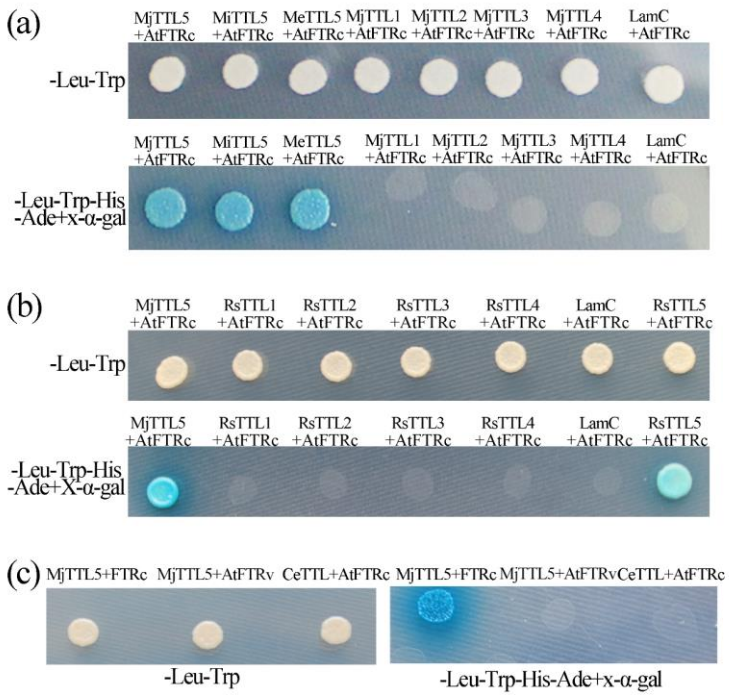

Fig. S14 Scrutinizing the interaction between AtFTRc homologs and MjTTL5 homologs. (a) Scrutinizing the interaction between different TTL homologs from *Meloidogyne* spp. and AtFTRc; (b) scrutinizing the interaction between different TTL homologs from *Radopholus similis* and AtFTRc; (c) scrutinizing the interaction between MjTTL5 and AtFTRv or CeTTL and AtFTRc. The coding sequence of MjTTL1-MjTTL5, RsTTL1-RsTTL5, MiTTL5 and MeTTL5 were separately cloned into the bait vector pGBK and co-transformed with the prey vector pGAD contained AtFTRc, and the bait vector pGBK containing MjTTL5 was co-transformed with the prey vector pGAD containing AtFTRv into the yeast strain AH109. pGBK containing LamC was co-transformed with pGAD containing AtFTRc as a negative control.

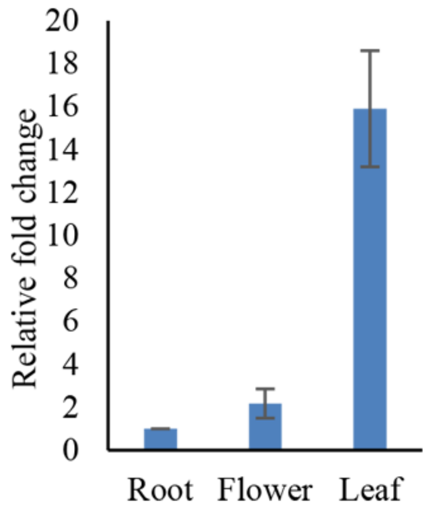

Fig. S15 Expression pattern of AtFTRc transcripts in multiple plant tissues. Total RNAs were extracted from leaves, flowers and roots from 30-day old wild type Arabidopsis. The AtActin gene (AT1G49240) was used as an internal control and the relative fold change was relative to the expression of roots. Error bars, ± SD.

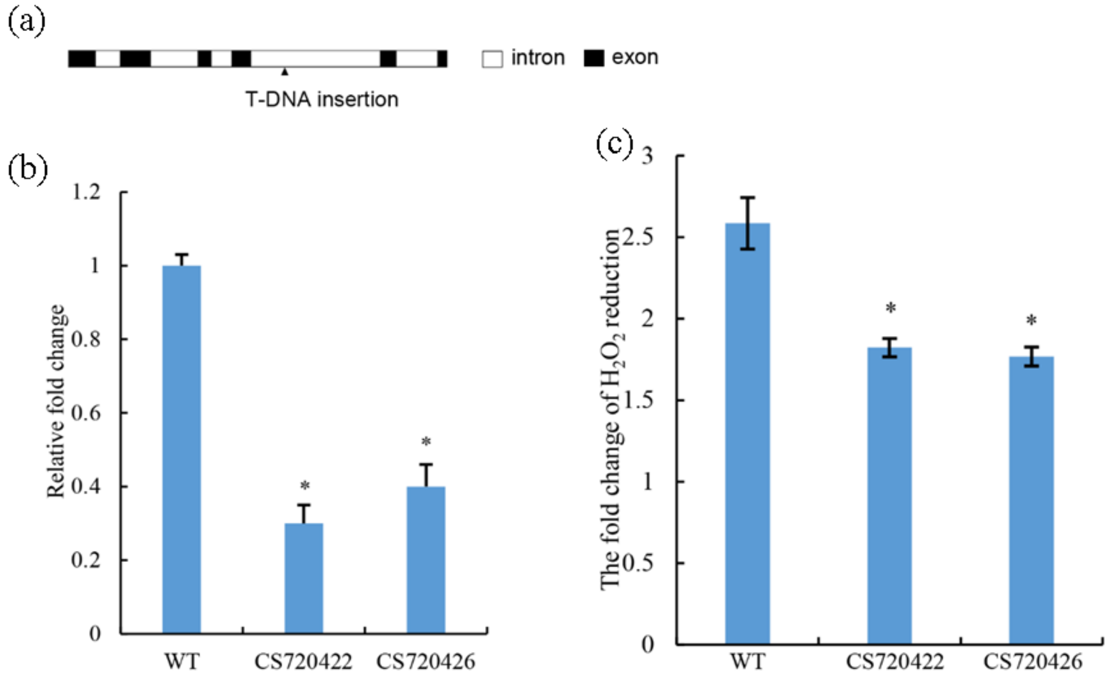

Fig. S16 Characterization of *AtFTRc* Arabidopsis mutants. (a) T-DNA insertion is located at the 4th intron of *AtFTRc* gene. (b) RT-qPCR assays of expression levels of *AtFTRc* in wild type (WT) and mutants. Roots were collected from 14-d-old plants for analysis. Fold change values were calculated using the $2^{-\Delta\Delta CT}$ method and presented as the change in mRNA level relative to that of WT. (c) The activity of MjTTL5 depends on AtFTRc. H_2O_2 reduction in WT was significantly higher than that in mutants, fold change was calculated by dividing the amount of H_2O_2 removed by Arabidopsis protein extracts in the presence of MjTTL5 with the amount of H_2O_2 removed by Arabidopsis protein extracts, the data was recorded at 10 min after H_2O_2 added. Data are presented as the means ± SD from five repeats, and asterisk indicates the values significantly different from WT as determined by *t-test* (P<0.05).

Methods S1 Protoplast isolation and transformation

Adapted from Lee *et al.* (2013) described method, details as follows:

1. Harvest the root tissues (*c.* 100 mg) from the plate and cut it to small pieces using scalpel;
2. Dip the tissues into 20 ml of enzyme solution (200 mg macerozyme R-10, 800 mg cellulose R-10, 1mg BSA, 10 µl mercaptoethanol, 400 mM mannitol, 8 mM $CaCl_2$, 5 mM MES, pH 5.6);
3. Incubate for 20–24 h with gentle agitation;
4. Filter the enzyme/ protoplast solution through the 140 µm mesh to remove undigested root tissues;
5. Centrifuge at 100 ***g*** for 10 min in a swinging-bucket rotor, discard the supernatant, and add pre cool 10 ml W5 buffer (154 mM NaCl, 125 mM $CaCl_2$, 5 mM KCl, 5 mM MES, pH 5.6) wash three times.
6. Keep the protoplast at 4°C for 1h;
7. Centrifuge the protoplast at 53***g*** for 5 min, and discard the W5 buffer, and add 200 µl MgMg buffer (400 mM mannitol, 15 mM $MgCl_2$, 5 mM MES, pH 5.6);
8. Add 30 µg of plasmid DNA into the protoplast;
9. Add 150 µl of PEG buffer (400 mM mannitol, 100 mM $CaCl_2$, pH 5.6, and 40% PEG-4000) into the protoplast, and mix gently;
10. Incubate at 28°C for 30 min;
11. Add 5 ml W5 buffer to wash three times;
12. Add 2 ml W5 buffer and resuspend the protoplast;
13. Incubate at 28°C for 16–24h.

Methods S2 The generation of constructs used in Protoplast transformation

AtNTRc was PCR amplified using the primers NTRcF/ NTRcR, mCherry was PCR amplified using the primers RFPpbiF/ RFPpbiR. The pBI121 vector which was digested by *Xba* I/ *Sac* I. 2 µl PCR product of AtNTRc, 2 µl PCR product of mCherry and 1 µl digestion vector were combined together, and add 5 µl pEASY-uni seamless cloning and assembly mixture (Transgen biotech Co., Beijing, China). MjTTL5 and AtFTRc were PCR amplified using the primers TTLuniF/ TTLuniR and FTRuniF/ FTRuniR. The pGFP vector was digested by *Bam*H I. 2 µl PCR product of MjTTL5 or AtFTRc, 1 µl digestion vector were combined together, and add 3 µl pEASY-uni

seamless cloning and assembly mixture, incubate at 50°C for 30 min, then transform into the DH5α competent cells. The scheme of the constructs were showed in the Fig. S16.

Methods S3 The generation of transgenic tomato roots expressing AtNTRc-mCherry

Adapted from Ron *et al.* (2014) described method, details as follows:

1. Seed sterilization and germination: place 30 seeds in an 50 ml tube; soak seeds in 10 ml of 50% commercial bleach (containing 0.1% Tween-20) for 25 min inverting the tubes every five min. Rinse three times with sterile ddH_2O; Plate seeds on B5 media; Incubate at growth chamber for 3–7 d (cotyledons are ready for transformation when they have expanded and the first true leaves have just emerged).
2. Preparation of *Agrobacterium rhizogenes* cells: Take a 100 µl aliquot of *A. rhizogenes* carrying AtNTRc-mCherry construct to start a 10 ml overnight culture in LB medium + antibiotic to O.D ≈ 0.5; Rinse *A. rhizogenes* cells three times with sterile ddH_2O; Dilute the *A. rhizogenes* cells to O.D. 0.1with sterile ddH_2O.
3. Cotyledon infection: dip the tomato cotyledons into the *A. rhizogenes* cells, and cut first the base off, and then cut the top off (two cut edges); 20-min later remove the liquid and blot explants dry on sterile Whatman filter papers; transfer onto solid B5 plates (1% sucrose, no antibiotics); co-cultivate for 24 h at 28°C covered from lights.
4. Root induction and selection: Rinse cotyledons three times with sterile ddH_2O (containing 500 mg l^{-1} cefotaxime); blot cotyledons dry on sterile Whatman filter papers; plated cotyledons bottom-side up on B5 + 3% sucrose + 500mg l^{-1} cefotaxime; incubate at 28°C until roots grow; Once roots are at least 1.0 cm long, roots were excised from the cotyledons and transfer to selection plate (B5 + 3% sucrose + 500 mg l^{-1} cefotaxime + 35 mg l^{-1} kanamycin).
5. The expression of AtNTRc-mCherry in tomato roots was confirmed by fluorescence microscope observation.

New Phytologist

References

Jaouannet M, Magliano M, Arguel MJ, Gourgues M, Evangelisti E, Abad P, Rosso MN. 2013. The root-knot nematode calreticulin Mi-CRT is a key effector in plant defense suppression. *Molecular Plant-Microbe Interactions* **26**: 97–105.

Lee MH, Lee Y, Hwang I. 2013. *In vivo* localization in Arabidopsis protoplasts and root tissue. In: Running MP, ed. *G Protein-coupled receptor signaling in plants: methods and protocols*. Totowa, NJ, USA: Humana Press Inc., 113–120.

Nam KH, Li J. 2004. The Arabidopsis transthyretin-like protein is a potential substrate of BRASSINOSTEROID-INSENSITIVE 1. *Plant Cell* **16**: 2406–2417.

Ron M, Kajala K, Pauluzzi G, Wang DX, Reynoso MA, Zumstein K, Garcha J, Winte S, Masson H, Inagaki S *et al*. 2014. Hairy root transformation using Agrobacterium rhizogenes as a tool for exploring cell type-specific gene expression and function using tomato as a model. *Plant Physiology* **166**: 455–469.
